# Geomicrobiology of a seawater-influenced active sulfuric acid cave

**DOI:** 10.1371/journal.pone.0220706

**Published:** 2019-08-08

**Authors:** Ilenia M. D’Angeli, Daniele Ghezzi, Stefan Leuko, Andrea Firrincieli, Mario Parise, Adriano Fiorucci, Bartolomeo Vigna, Rosangela Addesso, Daniela Baldantoni, Cristina Carbone, Ana Zelia Miller, Valme Jurado, Cesareo Saiz-Jimenez, Jo De Waele, Martina Cappelletti

**Affiliations:** 1 Department of Biological, Geological and Environmental Sciences, University of Bologna, Bologna, Italy; 2 Department of Pharmacy and Biotechnology, University of Bologna, Bologna, Italy; 3 DLR Institute of Aerospace Medicine, Radiation Biology, Köln, Germany; 4 School of Environmental and Forest Science, University of Washington, Seattle, WA, United States of America; 5 Department of Geological and Environmental Sciences, University of Bari “Aldo Moro”, Bari, Italy; 6 Department of Environment, Land and Infrastructure Engineering, Polytechnic University of Turin, Torino, Italy; 7 Department of Chemistry and Biology “Adolfo Zambelli”, University of Salerno, Fisciano (SA), Italy; 8 DISTAV, Department of Geological, Environmental and Biological Sciences, University of Genoa, Genoa, Italy; 9 HERCULES Laboratory, University of Évora, Évora, Portugal; 10 Instituto de Recursos Naturales y Agrobiologia, IRNAS-CSIC, Sevilla, Spain; The University of Akron, UNITED STATES

## Abstract

Fetida Cave is an active sulfuric acid cave influenced by seawater, showing abundant microbial communities that organize themselves under three main different morphologies: water filaments, vermiculations and moonmilk deposits. These biofilms/deposits have different cave distribution, pH, macro- and microelement and mineralogical composition, carbon and nitrogen content. In particular, water filaments and vermiculations had circumneutral and slightly acidic pH, respectively, both had abundant organic carbon and high microbial diversity. They were rich in macro- and microelements, deriving from mineral dissolution, and, in the case of water filaments, from seawater composition. Vermiculations had different color, partly associated with their mineralogy, and unusual minerals probably due to trapping capacities. Moonmilk was composed of gypsum, poor in organic matter, had an extremely low pH (0–1) and low microbial diversity. Based on 16S rRNA gene analysis, the microbial composition of the biofilms/deposits included autotrophic taxa associated with sulfur and nitrogen cycles and biomineralization processes. In particular, water filaments communities were characterized by bacterial taxa involved in sulfur oxidation and reduction in aquatic, aphotic, microaerophilic/anoxic environments (Campylobacterales, Thiotrichales, Arenicellales, Desulfobacterales, Desulforomonadales) and in chemolithotrophy in marine habitats (Oceanospirillales, Chromatiales). Their biodiversity was linked to the morphology of the water filaments and their collection site. Microbial communities within vermiculations were partly related to their color and showed high abundance of unclassified Betaproteobacteria and sulfur-oxidizing Hydrogenophilales (including *Sulfuriferula*), and Acidiferrobacterales (including *Sulfurifustis*), sulfur-reducing Desulfurellales, and ammonia-oxidizing Planctomycetes and Nitrospirae. The microbial community associated with gypsum moonmilk showed the strong dominance (>60%) of the archaeal genus *Thermoplasma* and lower abundance of chemolithotrophic *Acidithiobacillus*, metal-oxidizing *Metallibacterium*, *Sulfobacillus*, and *Acidibacillus*.

This study describes the geomicrobiology of water filaments, vermiculations and gypsum moonmilk from Fetida Cave, providing insights into the microbial taxa that characterize each morphology and contribute to biogeochemical cycles and speleogenesis of this peculiar seawater-influenced sulfuric acid cave.

## Introduction

Caves provide a unique portal into the deep subsurface habitat, which is typically characterized by relatively stable environmental conditions, absence of light and low nutrient supply. Several studies indicated that microbes sustain cave ecosystems by dominating primary production and fueling biogeochemical cycles [1, 2]. Chemolithotrophic microbial activities, which support chemosynthetic primary production in deep cave environments isolated from the external habitats, involve the oxidation of methane, manganese, iron, inorganic hydrogen, nitrogen, and sulfide [2–4]. Additionally, microbial cave life can also depend on small inputs of organic carbon, transported into the underground through percolating waters, air circulation and fauna. As the influx of organic carbon by these mechanisms is generally low and sporadic, most caves are oligotrophic [5]. To survive in these nutrient-poor environments, microorganisms typically organize themselves in collective structures, offering cooperation and mutualistic relationships and producing, as results of their interaction, biosignatures that can be observed within caves [5, 6]. In this context, underground environments have attracted wide attention because of the peculiar metabolic processes and microbial community structures featuring these oligotrophic ecosystems and because of the interesting mutual interactions established between microorganisms and minerals [4, 6, 7]. In particular, the importance of cell-mineral interaction was pointed out by the fact that biomineralization processes positively influence biofilm growth and microbial activity [8]. Several studies have demonstrated the strong influence of mineralogy and fluid composition on subsurface microbial diversity [3, 9]; in turn, the microbial activity has shown to have an impact on the mineral formations and cave speleogenesis [10,11].

Caves that have been formed by sulfuric acid speleogenesis (SAS) were shown to host various and peculiar biosignatures [4, 6, 12]. SAS caves can form both in confined (deep-seated) and unconfined (water table) conditions and are essentially related to the upwelling of acidic sulfide-rich waters that oxidize producing sulfuric acid (H_2_SO_4_) [see reaction (1)] [12–14]. In particular, the hydrogen sulfide (H_2_S) oxidation can occur: i) where deep and shallow water mix, or ii) where sulfidic water reaches the cave environment [10, 13]. SAS caves have been reported from many areas around the world and occur in carbonate rocks in different types of climates (from arid to tropical) [4]. The dissolution of carbonates caused by sulfuric acid [see reaction (2)] is rapid, and immediately induces the replacement of the host rock by gypsum and the release of CO_2_ into the environment.

$$H_{2}S+2O_{2}\to H_{2}SO_{4}$$ (1)

$${CaCO}_{3}+H_{2}SO_{4}+H_{2}O\to {CaSO}_{4}∙2H_{2}O+{CO}_{2}$$ (2)

The oxidation of H_2_S provides an important energy source for sulfur oxidizing microorganisms, which are able to sustain the microbial ecosystem in aphotic (cave) sulfidic environments, acting as primary producers and supporting growth not only of other microorganisms, but also of invertebrate and vertebrate animals [15, 16]. The biological oxidation of H_2_S also generates local acidity able to contribute to the dissolution of carbonate rocks, boosting cave speleogenesis [9]. Due to the high sulfur concentration, high temperature and oxygen-poor conditions, sulfidic caves are also considered analogues of environments prevalent early in Earth history or on other planets [6, 15].

Italy hosts 25% of all the known sulfuric acid caves worldwide, most of which are located along the Apennine chain [17]. Many of these are still active systems, in which rising sulfidic waters interact with fresh waters. Very well-known active caves are located in the Frasassi Gorge and Acquasanta Terme [18, 19], both of which host conspicuous microbial biofilms covering the sediments below the water table, visible as rock-attached streamers or sediment surface biofilms, and/or covering the cave walls featured by variable morphologies and colors such as viscous snottites, ragu-like deposits, and vermiculations [4]. Among these, the geomicrobiology of the water streamers has been the most extensively studied through molecular methods (16S rRNA clone library), microscopy and culture-based experiments [12, 15, 20] revealing the dominance of sulfur-oxidizing Gamma- and Epsilonproteobacteria and sulfur-reducing Deltaproteobacteria, the latter mainly occurring in the anaerobic community of the biofilm [21]. The morphology (i.e. long rock-attached streamers or shorter sediment biofilm) and microbial diversity of water streamers are mainly influenced by the water flow (shear stress) and the sulfide/oxygen ratio [22].

Fewer studies have investigated microbial communities growing on the walls of sulfidic caves. Among these, most of the studies investigated the geomicrobiology of snottites, which are extremely acidic biofilms clinging to overhanging gypsum cave walls or ceilings. They were described to host low microbial diversity dominated by members of the autotrophic and sulfur-oxidizing *Acidithiobacillus* genus, highly adapted to extreme acidity, and smaller populations of archaeal Thermoplasmatales and actinobacterial Acidimicrobiaceae groups [23–25]. On the other hand, vermiculations are discontinuous and irregular secondary mineral deposits, which are generally found on walls and ceilings of carbonic-acid caves [26]. They can exhibit different morphologies (i.e. dots, dendritic), colors (red, brown, grey, black) and dimensions [17]. Although the origin of vermiculations was initially associated with specific climatic and environmental conditions [27], some studies also suggested that biological activity could support their formation on the walls of sulfidic caves. In this case, they were named “biovermiculations”, [10, 12, 16, 28], in reference to their rapid generation and the intermediation of rich and active microbial populations [20]. Based on traditional bacterial cultivation methods and clone library analyses, that allow only a limited microbial description, this type of deposits was found to have a high microbial diversity.

In addition to vermiculations, bright white and soft deposits were also observed on walls and ceiling of sulfidic caves. These deposits are mainly composed of gypsum and are known as moonmilk, based on their texture [29]. These are distinguished from the more well-known calcite moonmilk deposits, which consists of microscopic crystals of different carbonate minerals (95%) with minor amounts of sulfates, silicates and phosphates (5%) [30, 31] or pure sulfates. Calcite moonmilk formation is often related to biological processes leading to either direct precipitation of calcite by microorganisms or mineral precipitation on microbial surfaces, functioning as nucleation sites [17, 32]. On the other hand, the microbial communities within gypsum moonmilk deposits, despite their abundance in sulfidic caves, have never been explored thus far.

**Fig 1:**
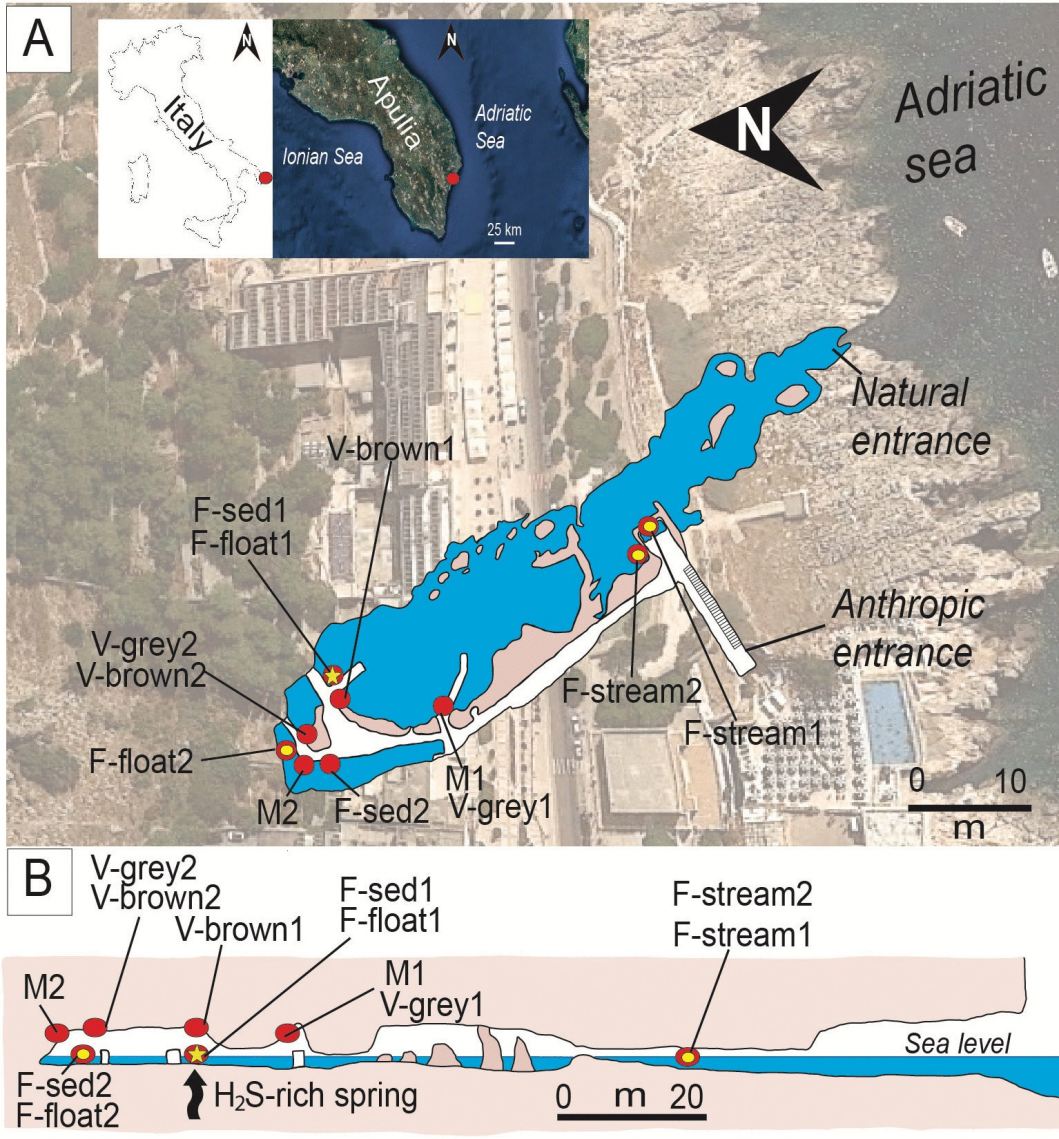
Location and plan-view maps of Fetida Cave A) Geographical location of Fetida Cave in Santa Cesarea Terme, Salento (SE Italy) and the cave map view from above. The cave opens along the Adriatic coastline following a NW-SE direction. B) Cross-section of Fetida Cave. In both A) and B), the sampling points are indicated as yellow points along the cave map. The yellow star (highlighted by an arrow in the cross-section) shows the position of the upwelling H_2_S-rich fluids.

**Fig 2:**
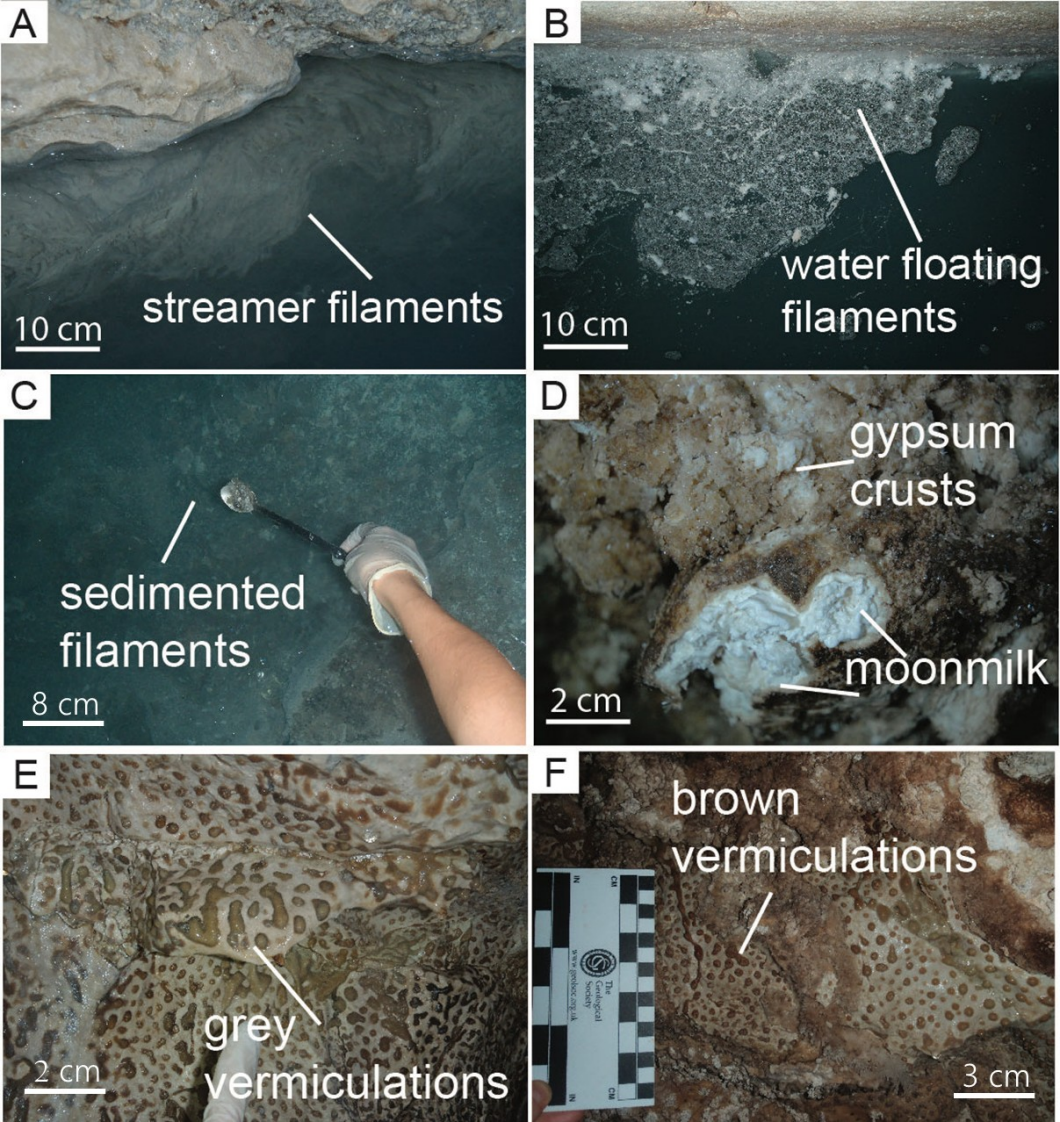
Field pictures of the most representative biofilms and deposits observed in Fetida Cave. A) Water filaments attached to the rock in the cave stream close to the entrance (named F-stream samples); B) Water filaments floating in the water in the inner zone of the cave (named F-float samples); C) Water biofilms sedimented on the water stream bed in the inner zone of the cave (named F-sed samples); D) Gypsum moonmilk deposits on the cave walls and ceilings (named M samples). It is surrounded by hard whitish gypsum crusts; E-F) Grey (named V-grey samples) and brown (named V-brown samples) vermiculations covering the cave walls and ceiling.

Fetida Cave (FC) is one of the four caves occurring along a 500-m long coastline in Santa Cesarea Terme, in the southeastern peninsular extension of Apulia (South Italy). All caves open at sea level at the foot of a limestone cliff (Fig 1) [33]. Santa Cesarea Terme and Capo Palinuro (Tyrrhenian coast South of Naples) represent unique examples of still active sulfuric acid cave systems, which are open at sea level [34]. Copious microbial communities are visible as biofilms/deposits in the deeper zone of the cave (in correspondence of the H_2_S-rich rising water). Biofilms occur in different morphologies such as white filaments or sedimented microbial mats in the water and vermiculation and moonmilk deposits on the cave walls and ceiling (Fig 2). In this work, the mineralogy, geochemistry and microbial diversity associated with the three different types of biofilms/deposits from Fetida Cave are described in order to provide a combination of geochemical and biological insights into sulfur-rich environments influenced by seawater.

## Material and methods

### Geological setting and Fetida Cave

From a geological standpoint, Apulia (Southeast Italy) represents a foreland basin (i.e. a weakly deformed area) characterized by a multilayered carbonate platform [35]. Santa Cesarea Terme (Salento area in Apulia) (Fig 1) is composed of a 1 km thick carbonate sequence of Cretaceous (100–66 Ma) to Middle Pleistocene (<0.781 Ma) age deposited above Upper Triassic (216–201 Ma) evaporites and dolostones, which are thought to be the main source of H_2_S.

Several caves are known along the Adriatic coastline, but the Fetida, Sulfurea, Gattulla, and Solfatara caves (from N to S) in the municipality of Santa Cesarea Terme are of special interest due to the upwelling of sulfidic waters used for human thermal treatments since the sixteenth century.

Fetida Cave (geographic coordinates: 40°02'04.3"N 18°27'20.6"E) is a 150 m long cave, partially submerged, and entirely carved in the “Calcari di Altamura Fm.” following a NW-SE regional lineament. It consists of a main cave stream characterized by the mixture of seawater and H_2_S-rich upwelling fluids. This stream originates from the discharging of a H_2_S-bearing spring (located in the inner part of the cave, Fig 1) into the seawater that enters the cave. The source of the H_2_S-rich fluids has not yet been identified, but is thought to derive from one of the following: i) deep-seated Triassic evaporites [29], ii) hydrocarbons from oil fields [36], iii) organic matter in the carbonate rocks themselves [37], iv) sulfate contained in seawater in contact with the organic matter within the Miocene calcarenites, or 5) complex interactions between heated seawater and deep-seated evaporite rocks [29].

Fetida Cave water is subject to a strong fluctuation due to seasonal changes, seawater movements and tidal fluctuations (of around ±0.7 m), which control the behavior of rising fluids and, consequently, the concentration of H_2_S in the cave atmosphere and water. The water level, at the present time, never reaches the upper walls and ceilings, which are mainly wetted by condensation and minor amounts of infiltration waters.

### Biofilm description and sample collection

Three types of biofilms and biogenic-like deposits (biodeposits) with different morphologies were collected from the water, walls and ceiling of Fetida Cave. This cave is located in a private land. Consequently, we asked and received the landlord’s oral permission to access the cave and collect the deposits/biofilms. In particular, the biofilms in the water can be categorized in white filaments floating on the water (named F-float samples), stream filaments attached to the rock (named F-stream samples) and biofilms sedimented on the stream bed (named F-sed samples) (Fig 2A–2C). Despite being more visible in the inner part of the cave (except for the F-stream samples), these whitish biofilms were generally sparsely distributed along the cave water stream depending on the sea water movement and tidal conditions. During low tides and calm seawater, representative samples of each type of filamentous structures were collected along the cave, at several distances from the sulfidic spring (Fig 1). A total number of six biofilms were collected along the cave length and were named F-float-1 and F-float-2, F-sed-1 and F-sed-2, F-stream-1 and F-stream-2 on the basis of their appearance (floating, sedimented or streamers). The latter two stream samples were collected at the cave entrance, as the whitish biofilms were commonly found on the two side walls of the water stream in this cave zone, while the sedimented and floating biofilms/filaments were more abundant inside the cave. F-sed-1 and F-sed-2 were collected at the bottom of the water stream (around 1 m below the water level).

In the inner part of the cave, two different types of biodeposits were observed on the walls and ceiling: i) elongated spotted biofilms of different colors (mainly grey and brown), which resemble the “biovermiculations” previously described by Hose et al. [28], and ii) bright white-colored deposits known as moonmilk. Moonmilk is used to describe a soft, wet, plastic, fine grained speleothem, which are typically found on cave walls [38]. Both vermiculations and moonmilk deposits copiously covered Fetida Cave walls and ceilings. While vermiculations were observed immediately above the water level (a.w.l), moonmilk deposits were solely found from 1 m a.w.l. upward, in areas where condensation was more abundant.

Samples of each biogenic-like morphotype were aseptically collected from different sites along the cave during two sampling campaigns (October 2015 and December 2017). The sampling sites and corresponding sample name are displayed in Fig 1, while the detailed description of the samples is provided in S1 Table. The samples representing the different deposits growing on the cave walls and ceiling were collected between 1.50 and 1.90 m of height along the boardwalk and were named V-brown or V-grey samples (V for vermiculations and grey or brown depending on the color), and M-1 and M-2 (M for moonmilk deposit) (Figs 1 and 2). All the collected samples of vermiculations and moonmilk deposits had a wet appearance and a pristine structure. Conversely, we avoided to sample dry vermiculations, as they were generally covered by thin gypsum crusts or crystals.

For the DNA extraction and microbial diversity analysis, subsamples were collected aseptically and transferred into falcon tubes filled with 5 ml of RNAlater. These were stored at -80°C until DNA extraction was performed. Aliquots of some subsamples were also used for elemental analysis and mineralogical characterization. Additionally, atmosphere and waters were periodically monitored at the cave entrance and in the inner cave zone (see below in “Geochemical analysis”).

### Geochemical analyses

Geochemical data of the cave water and air were obtained at the two major sampling locations i.e. at the cave entrance and in the deeper zone of the cave over two years (2015–2017, seven times in total) of ongoing research. Gases (O_2_, CH_4_, SO_2_, and H_2_S) were analyzed using a MSA Altair4x multigas detector (Pittsburgh, USA). The range of values and their resolution were 0–30 ± 0.1 vol% for O_2_, 0–100 ± 1% LEL for CH_4_, 0–20 ± 0.1 ppm for SO_2_, and 0–200 ± 1 ppm for H_2_S. The air temperature was measured with the silicon band-gap sensor loggers Niphargus (Natural History Museum Brussels, [39]) and Hobo (ONSET, [40]).

The concentration of S^2-^ dissolved in the water was analyzed in situ using the spectrophotometer Hach DR/2010 (Loveland, USA), whilst pH, T, TDS (Total Dissolved Solids) were monitored using Hanna HI991001 instrumentation (Padova, Italia). Na, K, Mg and Ca in water samples were measured by Atomic Absorption Spectrophotometry (AA-6800 Shimadzu, Kyoto, Japan). SO_4_^2-^, Cl^-^, F^-^, Br ^-^, PO_4_^3-^, NO_2_^-^, and NO_3_^-^ was measured with Ionic Chromatography (883 IC Plus Metrohm with high-performance separating column Metrosep A Supp. 5–250, Herisau, Switzerland). NH_4_^+^ was analyzed by Spectrophotometric determination using Indophenol Method (UV-VIS Recording Spectrophotometer UV-2501 PC Shimadzu, Kyoto, Japan), and alkalinity (HCO_3_^-^) through titration with H_2_SO_4_ 0.5N with automatic control of pH (809 Titrando Metrohm). Trace elements have been investigated using ICP-MS X SERIES 2 Thermo Scientific (Waltham, USA).

Calcite, dolomite, and gypsum saturation indices (SI) were calculated using the ratio between ion activity product (K_IAP_) and solubility products (K_sp_). Each K_IAP_ has been calculated using the Debye-Hückel equation to determine the ion activity coefficient. SI values close to 1 are indicative of saturated solution at equilibrium, whereas SI<1 are indicative of undersaturation (i.e., corrosive-dissolutive conditions).

Elemental analyses of the cave samples were carried out by oven-drying at 75°C, until constant weight was obtained, and by manually grinding them with a porcelain mortar and pestle. Total and organic C and N content were determined in triplicate using a CHSN-O Gas Chromatography Flash EA 1112 (Thermo Fisher Scientific Inc. Waltham, MA, USA). Organic C was measured removing carbonates using 37% HCl and distilled water (1:1 = v:v). Moreover, the concentration of Ca, Co, Cu, Fe, K, Mg, Mn, Na, P, and Zn was determined in triplicate with a PerkinElmer Optima 7000 DV ICP-OES. In particular, before the determination of chemical elements, samples (125 mg each) were subjected to microwave mineralization in a Milestone Microwave Laboratory System (mls 1200, Shelton, CT, USA) by a combination of 50% HF and 65% HNO_3_ (1:2 = v:v), and deionized water. Standard Reference Material was used to evaluate the analytical accuracy [41]. Indications on the pH values of the biodeposits were obtained in situ with litmus papers (range 0–14).

### Microscopy and Mineralogy

Samples for Field Emission Scanning Electron Microscopy (FESEM) were previously fixed with 2.5% glutaraldehyde in 0.1 M cacodylate-buffer (pH 7.4) at 4°C for 2 h and washed in cacodylate-buffer. Subsequently, they were postfixed in 1% osmium tetroxide for 1 h at 4°C and dehydrated by subsequent dilution series in ethanol and acetone finishing with 100% acetone before drying. The samples were then dried in a critical point drying device (Leica EM 300) at 34.5°C. Finally, the fixed samples were examined using a FEI TENEO microscope equipped with an Ametek EDAX detector for Energy Dispersive X-ray spectroscopy (EDX).

The mineralogical composition of moonmilk deposits and vermiculations was determined by using a Philips PW3710 diffractometer (current 20mA, voltage 40 kV, range 2θ 5–80°, step size 0.002° 2θ, time per step 2 sec) equipped with a Co-anode and interfaced with a Philips High Score software package for data acquisition and processing.

### DNA extraction, Illumina sequencing data and statistical analyses

Total DNA was extracted from all the samples using the DNeasy PowerSoil extraction kit (Qiagen) according to the manufacturer’s protocol, with some modifications described by Cappelletti et al. [42]. Briefly, 0.3 g of sample was employed, and DNA was extracted using a combination of bead-beading and lysis buffer with the addition of lysozyme and proteinase K. DNA was eluted into a final volume of 50 μL dH_2_O. The extracted DNA was used as template for PCR amplification targeting the V4 region of the 16S rRNA gene using the primers 515F (5’GTGCCAGCMG-CCGCGGTAA’3) and 806R (5’ GGACTACHVGGGTWTCTAAT’3) [43] modified with an Illumina adaptor sequence at the 5’ end. Samples were submitted to the Illumina MiSeq next-generation sequencing platform for indexing and pair-end sequencing (2x250 bp; reagent kit, v2) at the University of Graz (Austria). Reads were analyzed using the DADA2 package version 1.5.0 and workflow in R version 3.1.2 (http://www.R-project.org) [44]. Taxonomic assignment has been performed by querying the sequence reads against the SILVA SSU 128 reference database [45]. Diversity indices, richness estimations and Unifrac PCoA were analyzed using core-matrix-phylogenetic plugins on QIIME2 software [46]. Clustering and non-metric multidimensional scaling (nMDS) analyses were performed to visualize the similarity between the microbial communities using Primer-E v7 based on Bray-Curtis Distance Matrix [47]. Primer-E was also used to link environmental parameters to microbial community composition. The statistical significance of the correlation was assessed through Pearson coefficient analysis (with ρ>0.9 indicating significant correlation). The most abundant 16S rRNA sequences were aligned in the MEGA7 software [48] through the ClustalW algorithm and phylogenetic trees were constructed using the Maximum Likelihood method with 1000 bootstrap replicates. Sequencing data were deposited in the NCBI Sequence Read Archive (SRA) with the submission number PRJNA494546.

## Results

### Geochemistry of Fetida Cave

The concentration of O_2_ and SO_2_ in the atmosphere did not vary significantly along the cave, whereas the H_2_S concentration was higher in the inner zone of the cave (Table 1), reaching values of 15.4 ppm (S2 Table). Further, in the inner zone of the cave the atmospheric and water temperatures were more constant and slightly thermal (21–24°C) as compared to the cave entrance, S^2-^ concentration was also higher and pH was more acidic (Table 1, S1 Fig, S2 Table, S3 Table). Water samples collected inside the cave generally had lower concentration of Na, Cl^-^ and SO_4_^2-^ as well as of K, Mg and NO_3_^-^ as compared to the seawater samples collected along the coastline. Nevertheless, all the waters collected inside the cave could be classified as Na-Cl-SO_4_ waters (S2 Fig), due to the strong seawater influence. Seawater plays a key role in buffering the H_2_S-rich spring water to circumneutral pH in Fetida Cave. This aspect differentiates Fetida Cave from the far-from-the-coast freshwater sulfidic caves previously studied, in which the carbonate dissolution was reported to be the main driver in lowering acidity [10]. On the other hand, cave waters showed higher concentrations of Ca, Br^-^ and other trace elements (mainly Al, Mn, Fe, Zn, Ga, Sr, Cs, Ba) compared to seawater, except for Se and Tl (S4 Table). These results can be related to the mixing of the seawater with the H_2_S rich spring fluid which has the effect to dilute the seawater (decreasing the concentration of the most common seawater components i.e. Na, Mg). Comparing with the seawater, cave waters also showed lower values in saturation indexes (calcite, dolomite and gypsum). This indicates that rock/mineral dissolution phenomena are favoured increasing speleogenesis and cave enlargement (S2 Fig). In particular, Fetida Cave is actively undergoing sulfuric acid speleogenesis, during which H_2_S is oxidized to sulfuric acid in the subaqueous environment by microorganisms and subaerially on cave-wall surfaces due to H_2_S gas volatilization [10]. The sulfuric acid reacts with the limestone and the dissolution-replacement of the host carbonate rock releases Ca^+^, SO_4_^2-^ and CO_2_ as well as trace elements, such as Sr, Ba, Zn and/or insoluble Al- and Fe-oxy-hydroxides.

**Table 1:** Physico-chemical parameters of waters and air sampled at the entrance and in the inner zone of Fetida Cave and along the coastline^a^^,^^b^.

Cave Location	Sample	T °C	pH	O_2_ (%)	SO_2_ (μM)	SO_4_^2-^ (mg L^-1^)	S^2-^ (mg L^-1^)
Entrance	Air	21.4	ND	20.8	0.73	ND	0.3
	Water	23.0	7.4	ND	ND	3073.7	1.9
Inner zone	Air	22.8	ND	20.8	0.63	ND	2.6
	Water	25.1	7.0	ND	ND	2553.0	2.9
Outside (along the coastline)	Water	18.9	8.19	ND	ND	3003.1	BDL

^a^ Only mean values are reported. Tables S2 and S3 report the details (minimum and maximum values and standard deviations) of the physico-chemical analyses

^b^ ND = not determined, BDL = below the detection limit

These geochemical results indicate that the cave environment is strictly affected by two opposite influences i.e. the seawater effect entering the cave from the natural cave entrance and the H_2_S-springs arising and mixing with the seawater inside the cave. The sulfuric acid processes strongly depend upon environmental (i.e. tides) and climatic conditions (i.e. wave action). In general, moving from the entrance towards the deep zone of the cave, the marine influence decreases and the effect of rising acidic H_2_S-rich waters increases.

### Geochemistry and mineralogy of the cave samples

Water filaments, vermiculations and moonmilk deposits were distinguished by pH; indeed, white filaments have neutral pH (pH around 7), grey and brown vermiculations are slightly acidic (pH of 5–5.5), whereas moonmilk deposits are extremely acidic with a pH of 0–1 (Table 2). Furthermore, geochemical analyses showed that elemental composition, N and C content varied depending on the type of biodeposits, and, to a minor extent, among different samples representing the same type of biofilm (the mean values are reported in Table 2). In this respect, F-float samples generally displayed a higher abundance of N, organic C, Ca, Fe, Mn, P, Co, Cu as compared to F-stream samples. The most evident difference between F-sed and F-float samples was the concentration of inorganic C, which was more abundant in the sedimented biofilms as compared to the floating ones. On the other hand, F-stream samples were dominated by Na and Mg, elements that are mainly associated with the seawater composition, and are poor in N and C (Table 2).

**Table 2:** Physico-chemical properties and composition of the Fetida Cave biofilms and deposits.

Type of biodeposit	Main color	Mineralogy	T °C^a^	pH	N tot^b^	C org^b^	C inorg^b^	C tot^b^	C:N
Water stream filaments (F-stream)	White	ND	23.2 ±3.0	7.4	0.86 ±0.032	4.81 ±0.17	0.2 ±0.06	5.01 ±0.10	5.8
Filaments floating on the water (F-float)	White	ND	25.0 ±2.8	6.9	2.08 ±0.095	11.20 ±3.34	0.82 ±0.34	12.05 ±0.40	5.8
Sedimented water filaments (F-sed)	White	ND	24.2 ±2.1	6.9	0.51 ±0.025	3.75 ±0.40	5.57 ±0.14	9.32 ±0.31	18.3
Vermiculation (V-brown)	Brown	Quartz (SiO_2_), Diopside (CaMgSi_2_O_6_), Hematite (Fe_2_O_3_)	22.8 ±0.5	5–5.5	0.97 ±0.02	7.25 ±0.42	0.00	7.24 ±0.18	7.5
Vermiculation (V-grey)	Grey	Quartz (SiO_2_), Calcite (CaCO_3_), Muscovite [KAl_2_(Si_3_Al)O_10_(OH,F)_2_], Gypsum [CaSO_4_· 2H_2_O]	22.8 ±0.5	5–5.5	0.31 ±0.04	2.34 ±0.19	0.00	2.32 ±0.13	7.5
Moonmilk (M)	White	Gypsum [CaSO_4_· 2H_2_O]	22.8 ±0.5	0–1	0.00	0.14 ±0.05	0.04 ±0.37	0.19 ±0.01	-

^a^ Temperature values are those of water or air samples in correspondence with the sampling sites of filaments, vermiculations and moonmilk deposits.

^b^ Values are expressed in % dry weight. The mean values are presented for each type of biodeposit.

Moonmilk does not contain N and was generally poor in all the elements except for Ca (Table 2), which is mainly related to host rock dissolution. Brown vermiculations resulted to be richer in total N, organic C, Ca, Mg, Fe, Mn, P, Co, and Cu. In contrast, grey vermiculations showed the highest concentration in K (Table 3). These elemental differences could be associated with the mineralogical content of the wall/ceiling biodeposits (Table 2) which also explains the color differences among each biodeposit. For instance, brown vermiculations contain hematite, the greyish ones are rich in muscovite and quartz, whereas moonmilk is exclusively composed of gypsum (Table 2). The analysis of the C:N ratios was also carried out to evaluate the role of biofilms as food source in the ecosystem and compare these values with those reported in previous studies [16]. White filaments floating on the water or attached to the rocks were high-quality food source (C:N ratio of around 6), followed by vermiculations. A lower quality might be associated with the water biofilms/filaments sedimented on the stream floor.

**Table 3:** Chemical elements in the biodeposits from Fetida Cave^a^.

Sample	Na	K	Ca	Mg	Fe	Mn	P	Co	Cu	Zn
F-stream-1	231.41±19.32	43.87±0.89	1.79±0.20	5.48±0.54	0.04±0.01	0.00	0.27±0.09	0.07±0.02	1.37±0.22	0.00
F-stream-2	192.31±10.87	37.25±3.95	2.91±0.38	5.63±0.41	0.29±0.05	0.01±0.00	0.86±0.08	0.26±0.01	18.99±1.41	0.33±0.03
F-float-1	93.15±7.57	41.40±3.44	8.40±0.28	4.31±0.12	3.44±0.22	0.11±0.01	3.31±0.38	2.51±0.22	26.74±2.60	0.04
F-float-2	101.37±5.16	28.63±1.55	6.59±0.43	2.26±0.67	2.88±0.17	0.10	2.52±0.12	1.93±0.10	36.55±3.06	0.03
F-sed-1	60.42±7.09	28.13±5.09	25.04±1.42	2.26±0.67	2.14±0.18	0.12±0.01	1.23±0.15	2.05±0.32	30.63±4.92	0.10±0.02
V-brown-1	2.13±0.47	14.85±1.64	41.28±1.11	15.71±0.55	36.97±2.16	1.37±0.12	8.73±1.08	19.61±1.71	58.89±8.45	0.14±0.01
V-brown-2	1.81±0.41	10.53±0.04	51.13±4.77	7.49±0.73	19.14±0.10	0.84±0.06	9.24±0.93	9.50±0.48	51.42±4.04	0.09±0.01
V-grey-2	2.80±0.03	52.28±9.72	6.97±1.13	4.00±0.60	9.72±1.90	0.19±0.03	1.74±0.38	6.87±1.33	25.72±2.92	0.07±0.01
M-2	0.05±0.04	0.08±0.06	1.56±0.05	0.03±0.00	0.01±0.01	0.00	0.04±0.04	0.00	0.63±0.26	0.00

^a^ Concentration values are given as μg g^-1^, d.w.

### Microscopy observations

**Fig 3:**
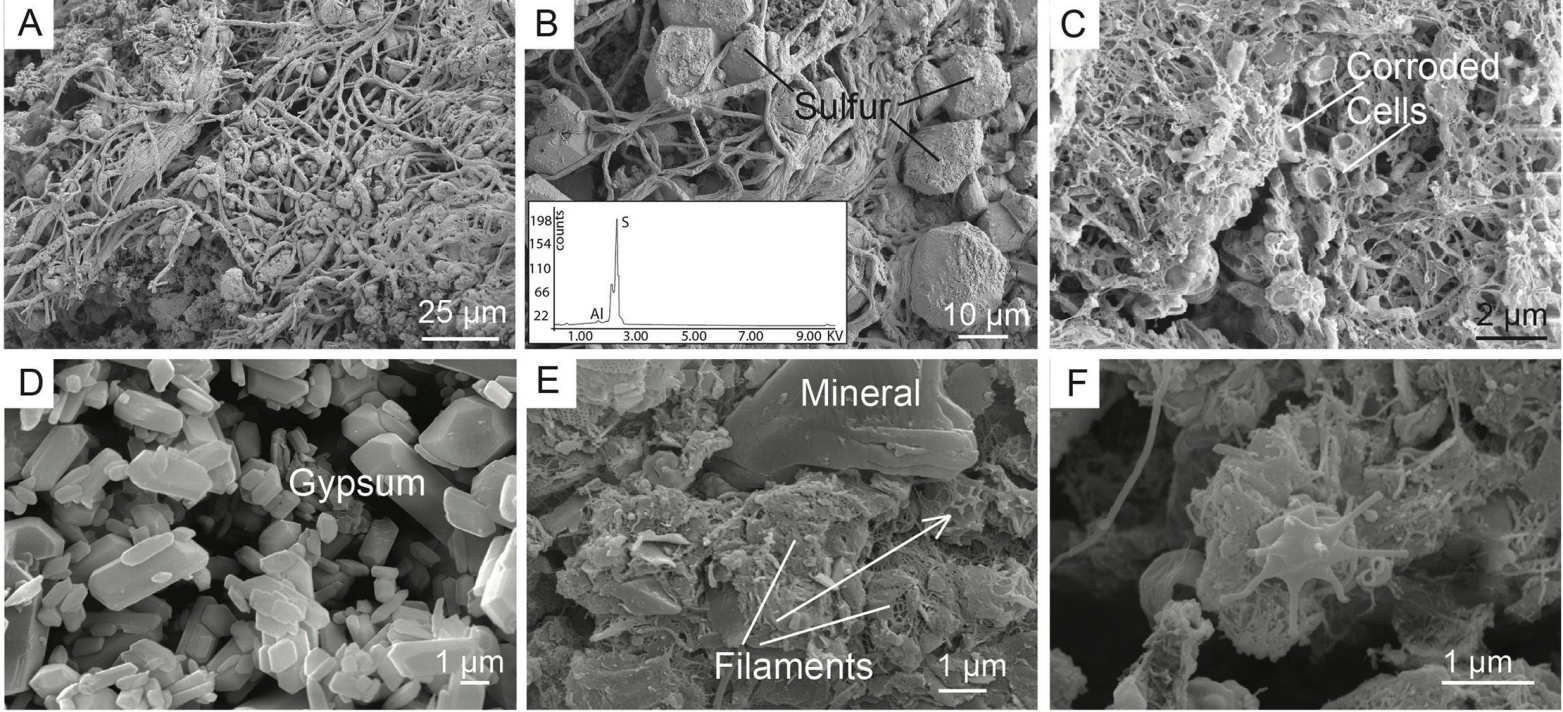
FESEM images of the biodeposits collected in Fetida Cave. A) Elongated filaments and particles characterized by sulfur crystals in F-stream samples; B) EDS spectrum of a sulfur crystal surrounded by filaments; C) F-sed collected from the water stream bed inside the cave. They appear more corroded than F-stream collected at the cave entrance; D) Gypsum microcrystals of moonmilk; E) V-brown is composed of a filamentous network, amorphous matrix and minerals. Minerals can be totally surrounded by filaments (see white arrow), which is probably the result of trapping and binding processes occurring in vermiculations; F) Prosthecate bacterium in grey vermiculation deposits.

FESEM was used to analyze the morphological differences of the three types of biodeposits found in Fetida Cave (Fig 3). In F-stream, intact filamentous biological structures of different diameters were visible intercut with abundant elemental sulfur particles, (Fig 3A and 3B). Filamentous structures were also visible in F-sed although they appeared generally thinner, organized in an intricate net that entraps coccoid cell-like structures with a partially corroded appearance (Fig 3C). This organization was similar to that observed in bacterial mats collected nearby a sulfidic spring in Capo Palinuro [49]. The observation of damaged microbial-like structures can be associated with the constant exposure of F-sed to rising acidic sulfidic water.

The moonmilk deposits are dominated by gypsum microcrystals (Fig 3D), whereas biological structures are rare. Vermiculations (both brown and grey) showed an amorphous (possibly extracellular) matrix entwined with interlocking filamentous structures (Fig 3E and 3F). Possible prosthecate bacteria were also visible in V-grey sample (Fig 3E); the latter being previously described from oligotrophic cave environments [50]. Mineral particles can be observed in Fig 3E. Such complex arrangement in vermiculations is linked to phenomena of trapping and binding of particles which are dispersed in the surrounding environment or subaerially transported. This capacity of entrapping particles would also explain the extraordinary presence of diopside in vermiculations (Table 2), a mineral generally absent in carbonate rocks, and possibly brought into Fetida Cave by wave action.

### Microbial diversity in Fetida Cave biodeposits

As a result of the processing of the demultiplexed fastq files with DADA2 package, 154,536 reads were obtained with an average length of 290 bp; they were clustered into a total of 2,969 sequence variances (SVs). Sequence variants are the DADA2 outputs that correspond to real amplicon denoised sequences. The SV analysis allows higher specificity and resolution as compared to operational taxonomy units (OTUs) [51]. Despite the high variation in number of reads obtained for each sample (S5 Table), the sequencing depth was high enough to describe the microbial communities in detail, as indicated by the shape of the rarefaction curves that reached a plateau (S3 Fig).

**Fig 4:**
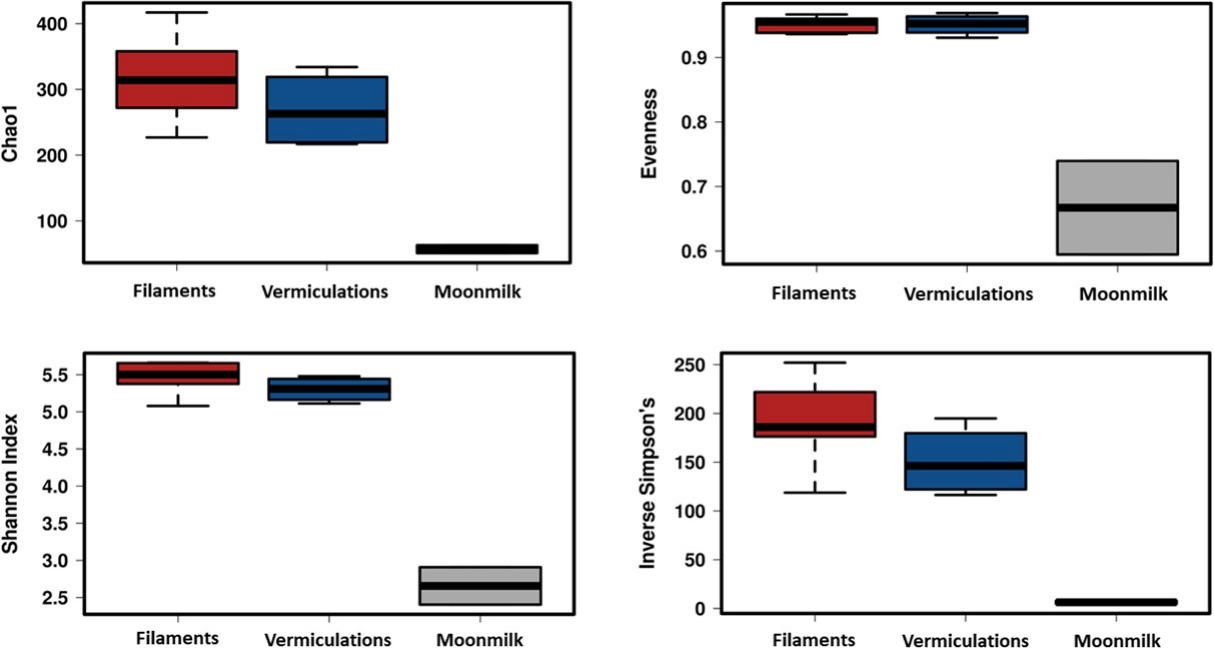
Diversity indices of the different biofilms from Fetida Cave. The three different box plots include the data corresponding to all the samples representing each biofilm/deposit. Water filament samples are reported in red, vermiculation samples are shown in blue, moonmilk deposit samples are grey.

Variability in SV richness (observed SVs and Chao1) among the samples was observed, with comparable values in relation to the sample type and location throughout the cave (S5 Table, Fig 4). The lowest richness was observed in moonmilk samples, showing a SV number up to six times lower than in the water filaments. The calculation of Shannon and Inverse Simpson Indices further confirmed the limited diversity pattern of moonmilk as compared to vermiculations and white filaments.

**Fig 5:**
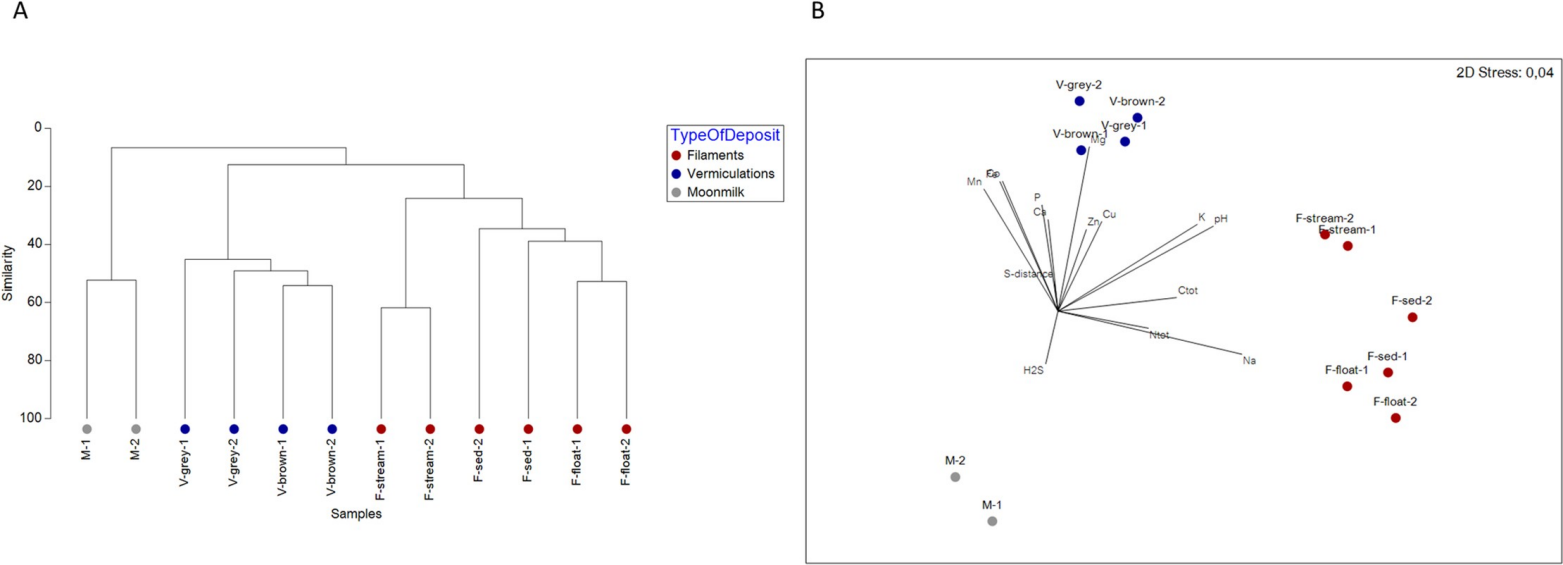
Clustering of the different biofilm samples from Fetida Cave Cluster. A) Clustering calculated using the Bray-Curtis distance between samples based on SV taxonomy classification in SILVA. B) Non-metric multidimensional scaling (nMDS) showing correlation between physico-chemical factors and biofilm microbial community composition. Water filaments are represented in red, vermiculations in blue, and moonmilk deposits in grey.

A very low number of SVs were shared among samples (S4 Fig); nevertheless, the clustering, on the basis of the SV taxonomy, revealed (Bray-Curtis) distance values ranging between 40 and 65% within each biofilm group (Fig 5A). In particular, the samples clustered based on the substrate: 1) water for filaments (F-float, F-sed, F-stream) and 2) cave wall/ceiling for moonmilk and vermiculation deposits (M, V-grey, V-brown). Within each group, F-stream samples clustered apart from F-sed and F-float samples, and moonmilk grouped apart from the vermiculations, being the latter further sub-clustered depending on the color (grey or brown). The non-metric multidimensional scaling (nMDS) analysis confirmed the clustering of Fetida Cave samples, mainly by type of biodeposits, and, in the cases of water filaments, by morphology and/or sampling location, as both F-stream groups were collected at the cave entrance (Fig 5B). Among a selection of physico-chemical parameters examined in this work (i.e. pH, H_2_S, N_tot_, C_tot_, and the elements in Table 3), pH, Na, K and Mg strongly correlated (p > 0.9) with the microbial diversity of the three biofilms/deposits. These results indicate that biofilm microbial communities were most influenced by pH in moonmilk, seawater in water filaments (Na and K) mineralogy in vermiculations (richness of Mg in V-brown). The concentration of Fe, Co, and Mn also affected the microbial diversity, albeit with a lower Pearson correlation (ρ>0.4).

### Microbial community composition in Fetida Cave biodeposits

**Fig 6:**
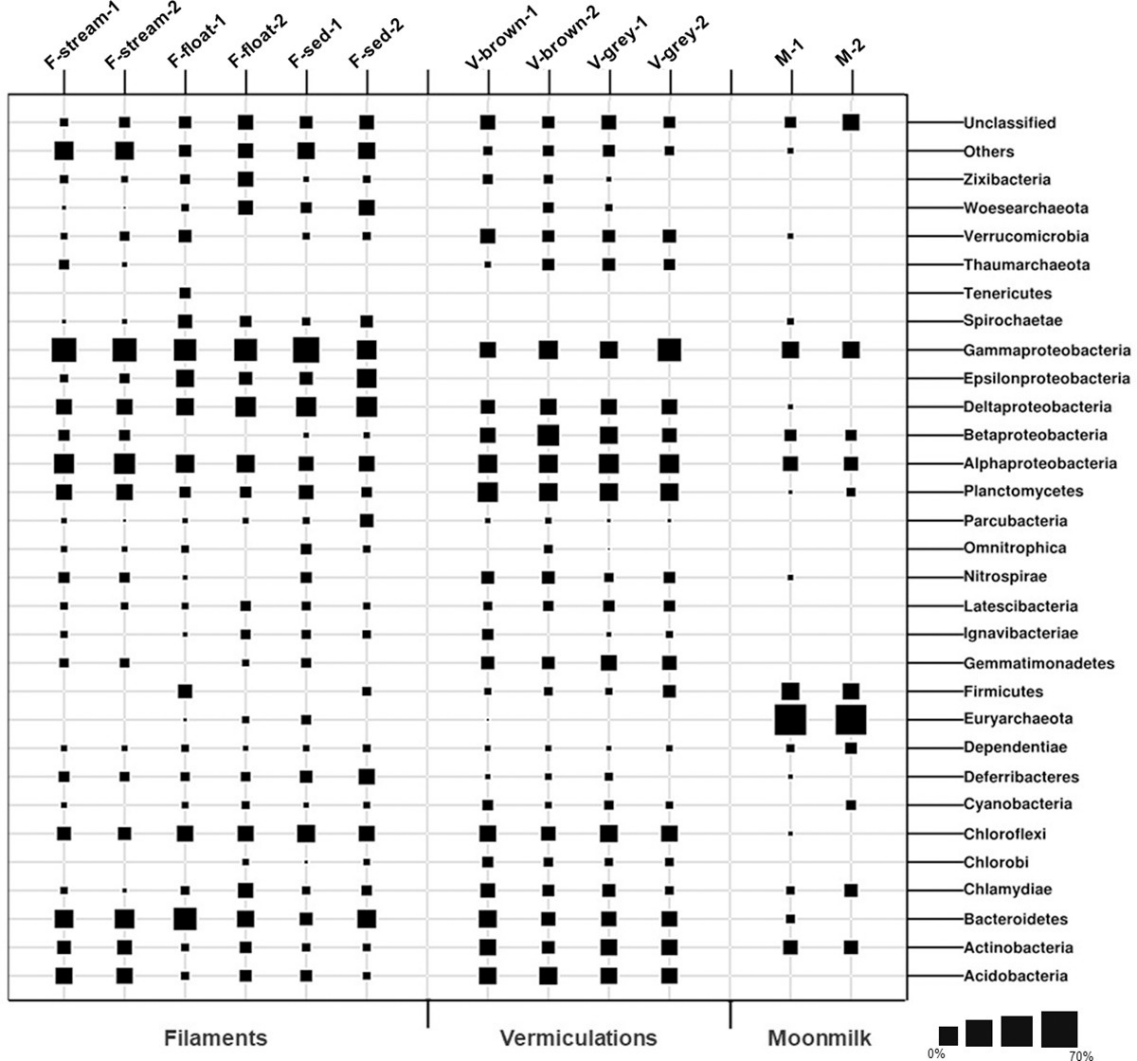
Microbial community composition at phylum/proteobacteria class level of Fetida Cave samples representing water filaments, vermiculations and moonmilk deposits. Microbial phyla and proteobacterial classes with abundance <1% are included in “Others” (S1 File).

A total of 47 bacterial phyla (with abundance >1%) were identified in the different Fetida Cave biofilms, of which 22 phyla were detected in water filaments, 18 phyla in vermiculations and only 7 phyla in gypsum moonmilk (Fig 6). Around one third of the SVs were unclassified at taxonomy levels lower than phylum in vermiculations and water biofilms, indicating the high presence of unexplored microbial taxa. This percentage was lower for moonmilk SVs (7–12%) (S1 Appendix).

#### Biofilms in the water

Bacteria dominated all the analyzed water biofilms. Archaeal sequences accounted for a maximum of 6% in F-float samples and were mainly affiliated with Woesearchaeota phylum (unclassified at lower taxonomic levels) (Fig 6).

**Fig 7:**
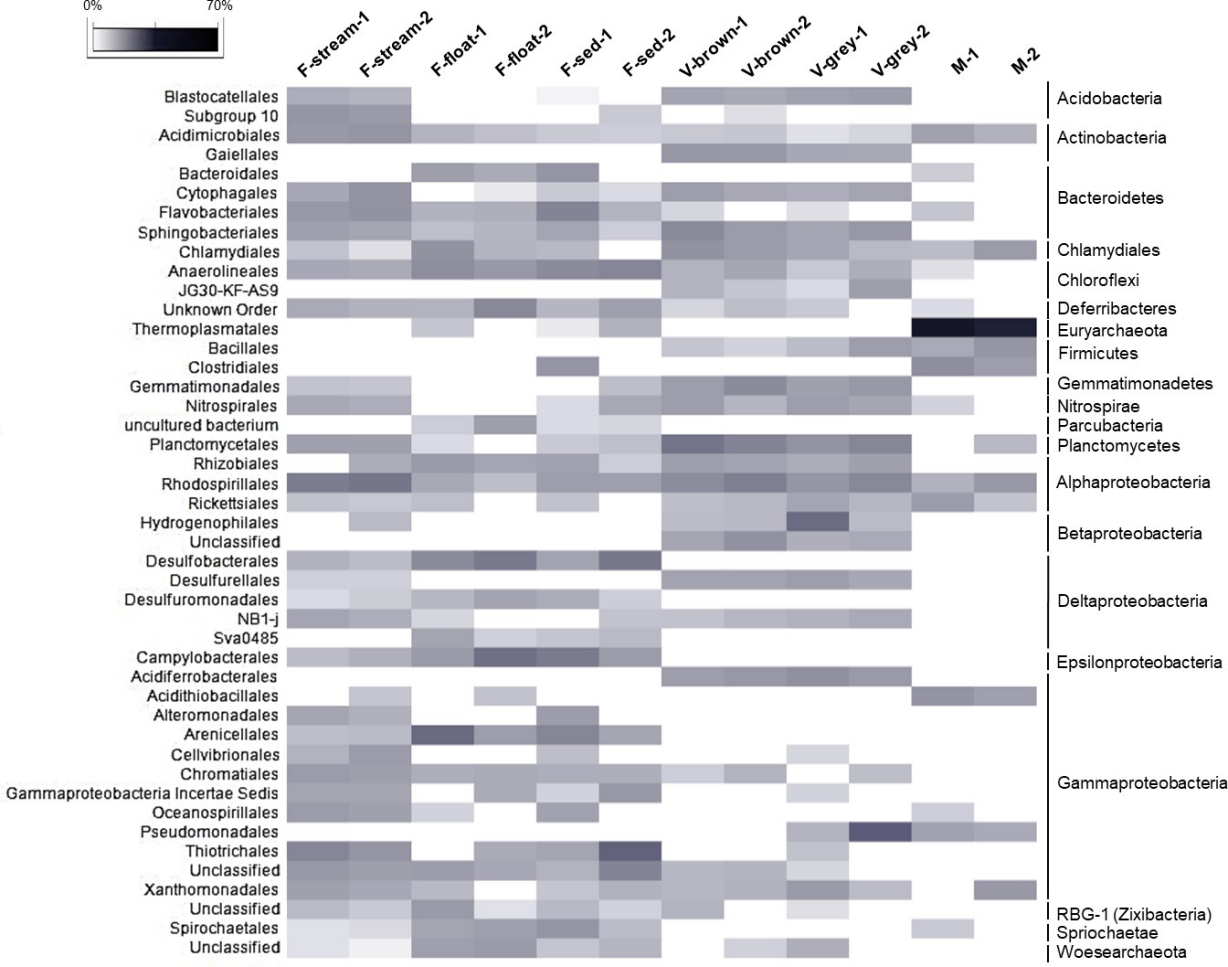
Heatmap showing the abundance of the microbial orders present in Fetida Cave samples. Only orders with abundance >2% in at least one sample are reported. The higher taxonomy affiliation of the orders is reported on the right side of the figure.

**Fig 8:**
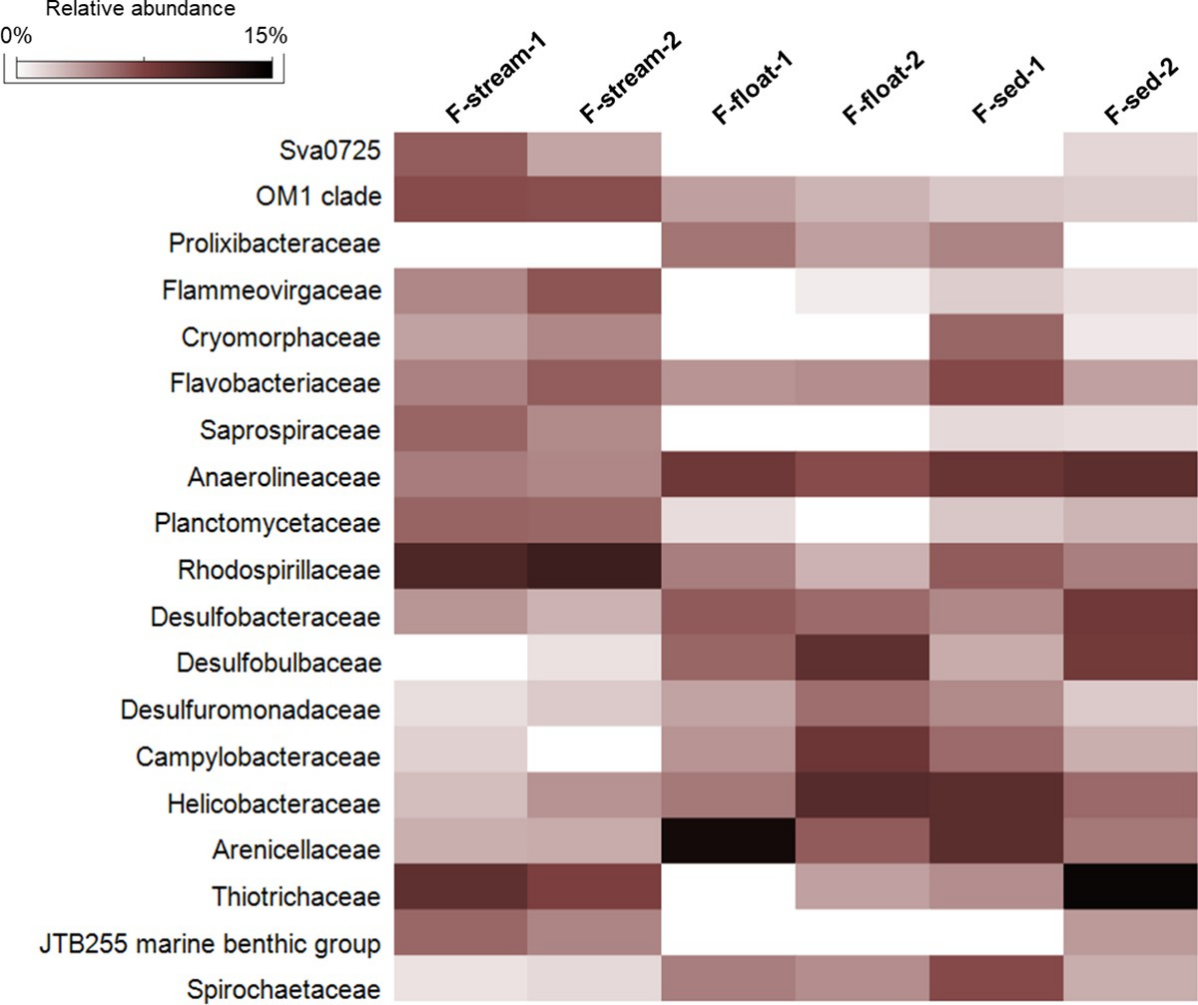
Heatmap showing the abundance of the microbial families present in Fetida Cave water filaments. Only those families with a relative abundance >2% are shown.

The phylum Proteobacteria was the most abundant in all the water biofilms (abundance of 47–60% of the bacterial sequences). In particular, F-stream showed the highest abundance of Gammaproteobacteria class (24–27% of each microbial community) followed by Alphaproteobacteria (13–15%, almost exclusively of Rhodospirillales order of Rhodospirillaceae family) (Figs 6 and 7). In these samples, classified Gammaproteobacteria sequences were mostly affiliated to Thiotrichales (of Thiotricaceae family), Chromatiales and Oceanospirillales orders (Figs 7 and 8). Bacteroidetes (10–12%, mainly constituted by Cytophagales and Flavobacteriales) was the second most abundant phylum after Proteobacteria, followed by Acidobacteria (6–7%, of Subgroup 10 and 4), Planctomycetes (6–6.5%), and Actinobacteria (3.5–4.5%, mainly of Acidimicrobiales order) (Figs 6 and 7).

F-float and F-sed samples were generally dominated by Gammaproteobacteria (12–34% of each microbial community) and Deltaproteobacteria (8–15%), followed by Alphaproteobacteria (4–10%, mainly of Rhizobiales and Rhodospirillales orders) and Epsilonproteobacteria (3–12%) (Figs 6 and 7). At lower taxonomy level, among Gammaproteobacteria, Arenicellales (of Arenicellaceae family) was abundant in F-float and F-sed (13% and 6.5%, respectively) and Thiotricales (of ‘*Candidatus* Thiopilula*’* genus) was predominant (12.5%) in F-float-2 (Figs 7 and 8, S6 Table). Deltaproteobacteria was mainly composed of Desulfobacterales and Desulfuromonadales orders, in particular, of *Desulfobulbus*, *Desulfocapsa*, MSBL7 genera, and Desulfuromonadaceae family. Epsilonproteobacteria sequences were exclusively related to Helicobacteraceae and Campylobacteraceae families which were mostly represented by the sulfur- and sulfide-oxidizing genera *Campylobacter*, *Sulfurimonas*, *Arcobacter*, *Sulfurovum* (Fig 8, S1 Appendix). Bacteroidetes (mainly belonging to Flavobacteriales and Bacteroidales orders) was highly variable among F-sed and F-float samples and ranged between 3 and 21%, representing the most abundant phylum in F-sed-1 sample (Figs 6 and 7). Chloroflexi accounted for 6–9% of the F-sed and F-stream microbial communities and mainly included members of Anaerolineaceae family (Figs 6 and 8).

By comparing the microbial diversity of F-stream to F-float and F-sed, gammaproteobacterial communities were different at low taxonomic level (Fig 7) and microbial communities of F-float and F-sed were generally enriched, as compared to F-stream, in Deltaproteobacteria, Epsilonproteobacteria, and Chloroflexi. On the other hand, F-sed and F-float had lower abundance of Acidobacteria, Actinobacteria, and Planctomycetes (Figs 6 and 7). The presence of sequences affiliated to Chlamydiae and Spirochaetae was also higher in these samples as compared to F-stream, although they never exceeded 5% abundance (Figs 7 and 8). All the water filaments (F-stream, F-sed and F-float) showed abundance >1% of *Caldithrix* genus (Deferribacteres phylum), reaching a maximum presence of 6.2% in F-sed-2.

**Fig 9:**
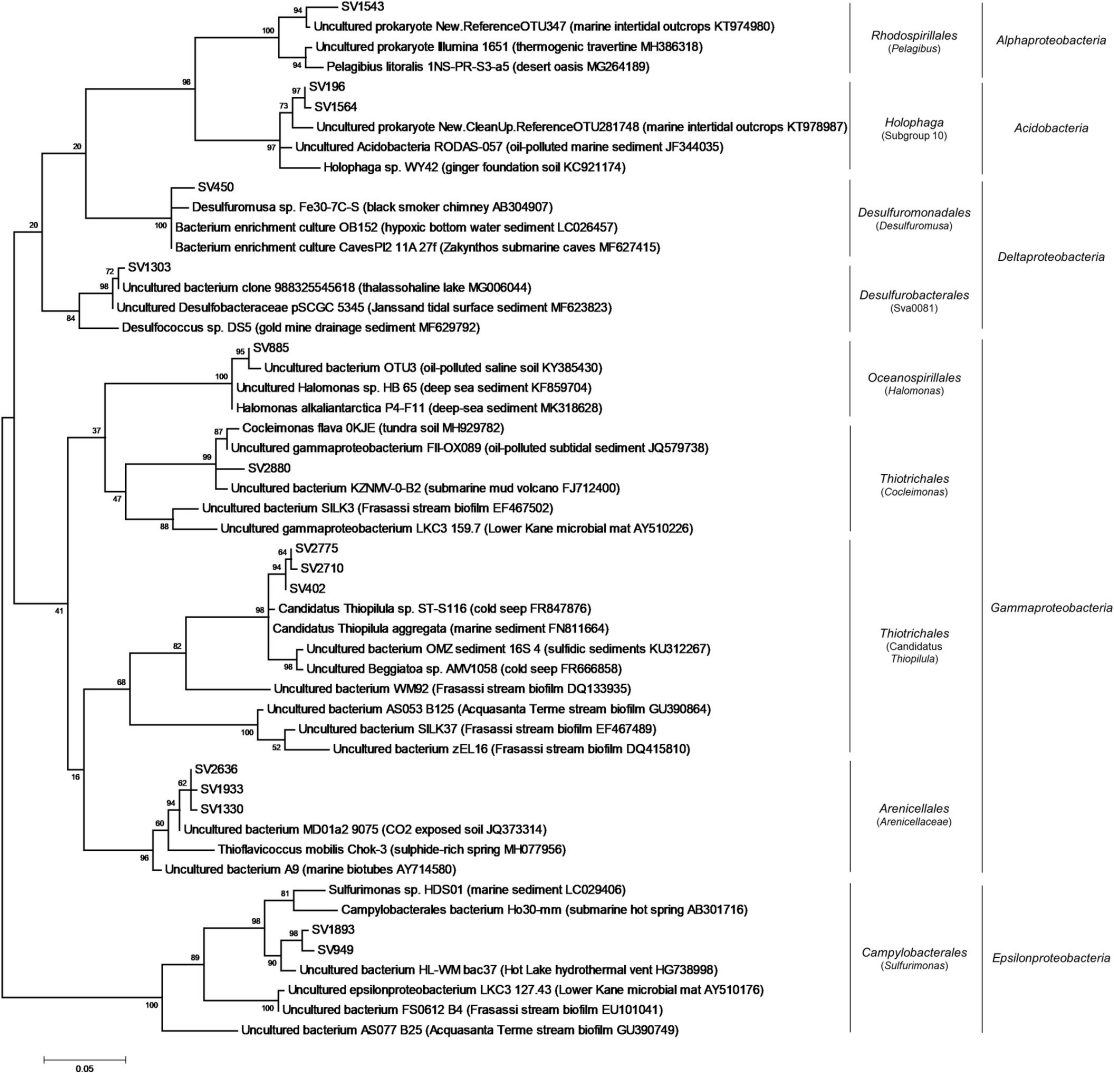
Phylogenetic tree of the most abundant SVs within water filaments/biofilms from Fetida Cave. For taxonomy details and Best Blast Hit of the SVs reported, see S6 Table.

The phylogenetic analysis of high abundant SVs identified in Fetida Cave water filaments showed their affiliation with sequences retrieved from tidal and submarine sediments, also exposed to elevated CO_2_ concentration or oil pollution, hypersaline lakes, and deep-sea environments loaded with energy-rich chemicals (e.g. cold seeps, hydrothermal deep vents) (Fig 9, S7 Table). On the other hand, only a few SVs showed affiliation with sequences retrieved from analogous filaments described in other sulfidic caves (e.g. Frasassi Cave). In this regard, the SVs related to ‘*Candidatus* Thiopilula’ and *Sulfurimonas* showed a low similarity (<90%) with the closest database sequences affiliated to Thiotricales and Campylobacterales, respectively, retrieved from water filaments collected from Frasassi and Acquasanta Terme caves [15].

#### Vermiculations

**Fig 10:**
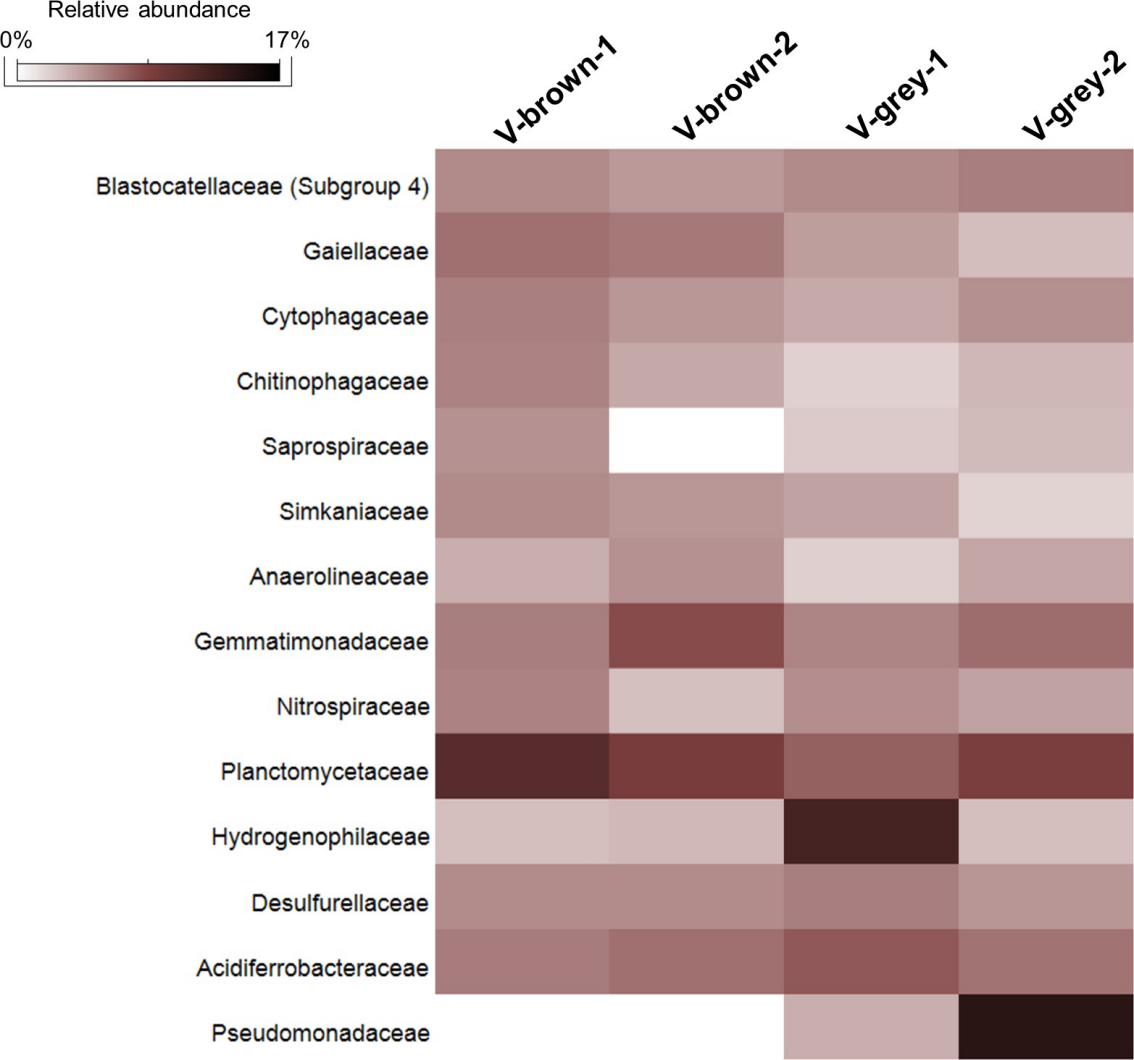
Heatmap showing the abundance of the microbial families present in Fetida Cave vermiculations. Only those families with a relative abundance >2% are shown.

*Bacteria* were predominant in all the vermiculations, while archaeal sequences constituted <4.3% of the total sequences, being mainly composed of Thaumarchaeota phylum (included in “Others” in Fig 7 and S1 File). The bacterial populations of the FC vermiculations were mainly composed of Proteobacteria (44–46% in V-grey samples and 26–36% in V-brown samples), followed by Planctomycetes (9–13%, mainly of Planctomycetaceae family), Acidobacteria (6–9%, with Blastocatellales Subgroup 4 as the most abundant classified family), Chloroflexi (4–9%, mainly of Anaerolineales and JG30-KF-AS9 orders), Bacteroidetes (4–9%, mainly of Cytophagales and Sphingobacteriales orders), Actinobacteria (3–7%, with *Gaiella* genus mainly present in the two V-brown samples), and Nitrospirae (1–3%, mainly represented by *Nitrospira* genus) (Fig 10, S1 Appendix). At class level, Alphaproteobacteria and Deltaproteobacteria showed a quite uniform abundance in the different vermiculation samples, ranging between 10 and 13% and between 4 and 7%, respectively (Fig 6). Alphaproteobacterial sequences were mainly affiliated to members of Rhizobiales and Rhodospirillales orders, unclassified at lower taxonomic levels (Fig 7). Around half of the deltaproteobacterial sequences were affiliated to Desulfurellaceae family (Figs 7 and 10). Conversely, Betaproteobacteria and Gammaproteobacteria showed more variation among vermiculations, each representing the largest taxonomic group of either V-grey-1 or V-grey-2, respectively (Fig 6). Most of betaproteobacterial sequences belonged to unclassified taxa, except for those affiliated to Hydrogenophylales order, and in particular, to the sulfur-oxidizing *Sulfuriferula* genus in V-grey-1 (Fig 7, S7 Table, S1 Appendix). On the other hand, in all the vermiculation samples almost half of the Gammaproteobacteria sequences were affiliated to *Sulfurifustis* genus of Acidiferrobacteraceae family (Fig 10, S1 Appendix). The two grey vermiculation samples shared the presence of Pseudomonadaceae family that was absent in the brown vermiculations. In particular, *Pseudomonas* represented 17% of the microbial population of V-grey-2 (Fig 10, S1 Appendix).

**Fig 11:**
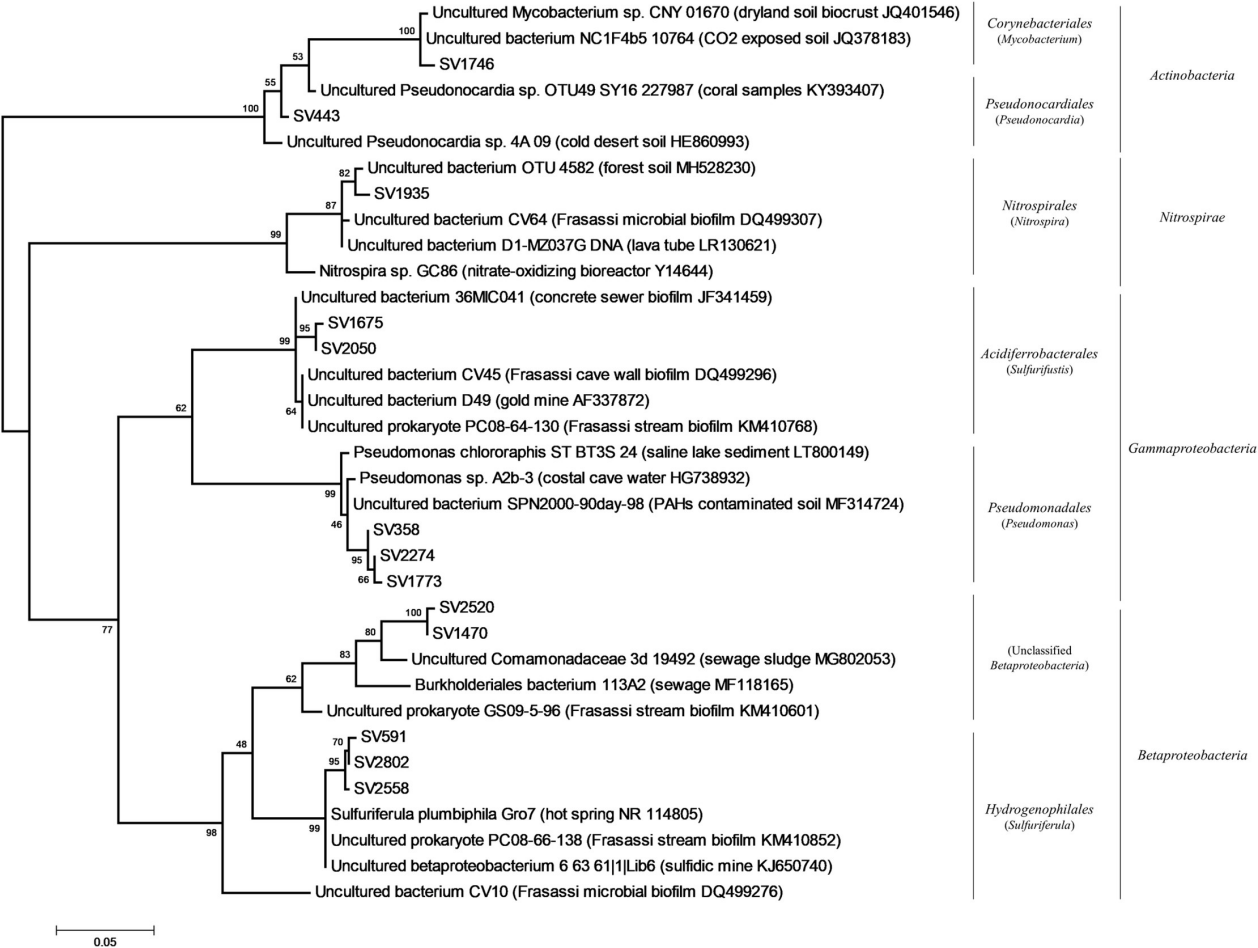
Phylogenetic tree of the most abundant SVs in V-grey (colored in grey) and V-brown (colored in brown) samples. For taxonomy details and Best Blast Hit of the SVs reported, see S7 Table.

At SV level, the most abundant SVs were affiliated to different bacterial taxa that were specific to each V sample (S7 Table, S1 Appendix). In particular, the most abundant SVs were affiliated to *Sulfuriferula* and *Sulfurifustis* genera in V-grey-1, to *Pseudomonas* genus in B2-Vg and to taxonomically undefined members of Betaproteobacteria in V-brown-2. Conversely, in V-brown-1, several SVs belonging to different bacterial taxa (e.g. *Opitutus* of Verrucomicrobia, *Mycobacterium* and *Pseudonocardia* of Actinobacteria) were present at ~1% without showing a clear dominance (S7 Table, S1 Appendix). Phylogenetic analysis of the most abundant vermiculation SVs indicated their affiliation with sequences retrieved from extreme environments (e.g. desert, dry lands, high CO_2_ exposed soil), mine tailings, metal rich sediments and concrete corrosion due to microbial activities in H_2_S-rich wastewater (Fig 11, S7 Table), possibly the first being related with the harsh conditions of the cave wall as growth substrate and the second with the high concentration of microelements and metals featuring this type of deposit (Table 3). Some SVs were also affiliated with sequences retrieved from vermiculations and water biofilms collected from a Frasassi Cave zone (Pozzo dei Cristalli) characterized by slowly flowing and stagnant pools [16].

#### Moonmilk

**Fig 12:**
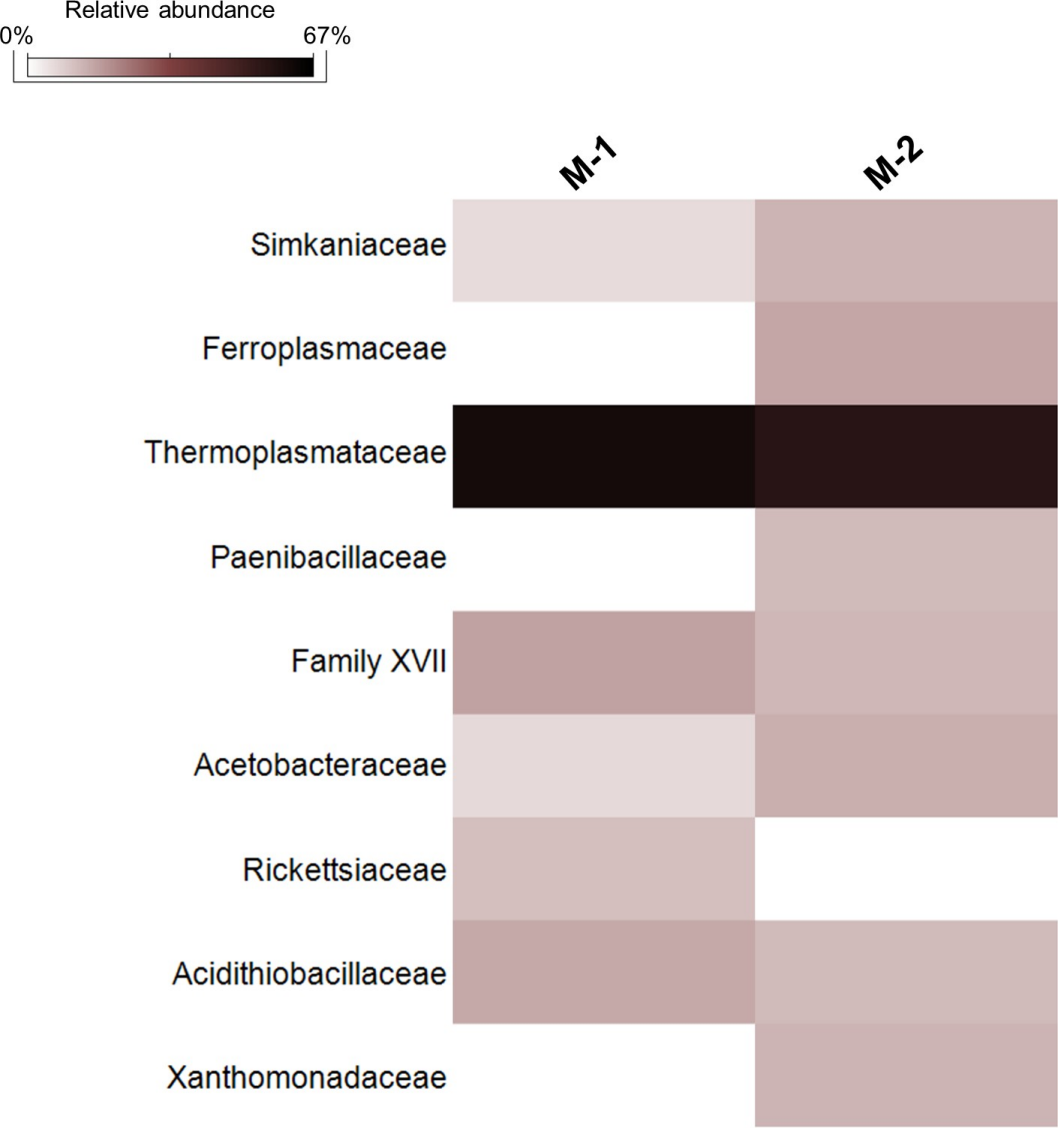
Heatmap showing the abundance of the microbial families present in Fetida Cave moonmilk deposits. Only those families with a relative abundance >2% are shown.

Moonmilk was dominated (61–67%) by Archaea-related sequences exclusively belonging to the Thermoplasmatales order of Euryarchaeota phylum (Fig 7, S1 Table). In M-1, they were only represented by *Thermoplasma* genus of Thermoplasmataceae family, whereas in M-2, in addition to *Thermoplasma*, a few Thermoplasmatales-related sequences were also affiliated to *Ferroplasma* genus (Figs 6, 7 and 12, S1 Appendix). Among the bacterial sequences, Gammaproteobacteria and *Firmicutes* accounted for 7–8.5% in each M sample (Fig 6). In particular, *Acidithiobacillus* was the most representative genus of Gammaproteobacteria in both M samples, whereas, Xanthomonadales *Metallibacterium* was highly abundant only in M-2 (Fig 7, S1 Appendix). Pseudomonadales were 1–2.5% in both the M samples. *Firmicutes* were almost exclusively composed by members of Bacillales order, mainly of Paenibacillaceae and *Acidibacillus* genera, and *Sulfobacillus* of the Clostridiales order (Fig 12, S1 Appendix). At a lower abundance, Rhodospirillales, Rickettsiales and Chlamydiales were present in both M samples, as well as Actinobacteria Acidimicrobiales and Corynebacteriales orders (Fig 7).

**Fig 13:**
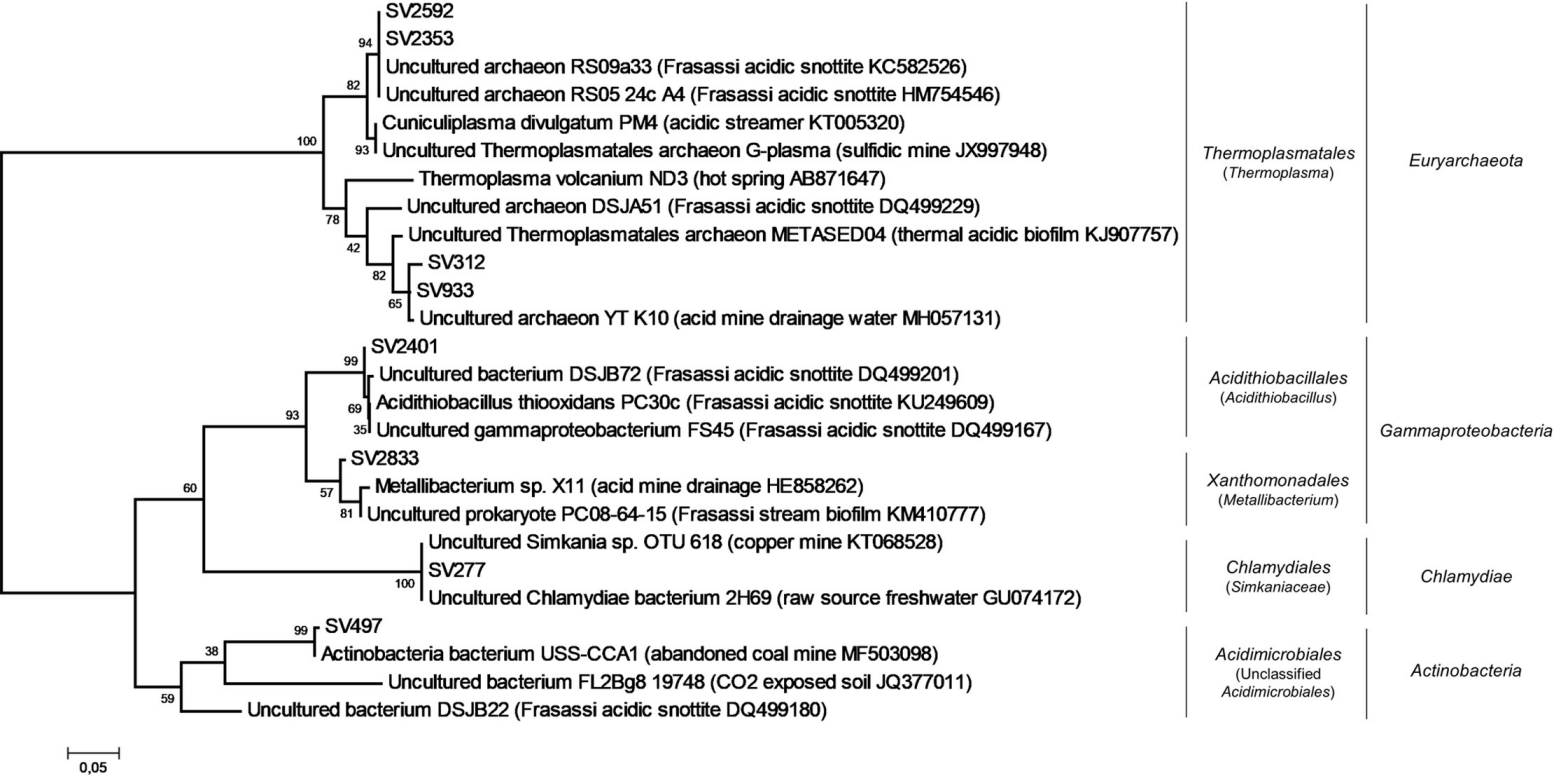
Phylogenetic tree of the most abundant SVs in gypsum moonmilk in Fetida Cave. For taxonomy details and Best Blast Hit of the SVs reported, see S8 Table.

The two most abundant SVs (SV2592 and SV2353) identified in the two gypsum moonmilk samples in FC were also shared by them (S4 Fig), being both affiliated to *Thermoplasma* genus and highly similar (99%) to sequences retrieved from Frasassi Cave acidic snottites (S8 Table, S1 Appendix). Additional abundant SVs were affiliated with members of *Acidithiobacillus*, *Metallibacterium*, of the Simkaniaceae and Acetobacteraceae families, respectively (Fig 12, S8 Table, S1 Appendix) and, at a lower level, with other acidophilic bacterial taxa i.e. Acidimicrobiales order, *Acidibacillus* and *Acidothermus* genera (S1 Appendix). In the phylogenetic tree, the moonmilk SVs were mostly related with sequences retrieved from acidic environmental sites including acidic cave biofilms (snottites) described from Frasassi Cave, mine-associated deposits or acid drainage waters (Fig 13, S8 Table).

## Discussion

The present study investigates the geochemistry and microbiology of three types of biodeposits (water filaments, vermiculations and moonmilk deposits) found in Fetida Cave, a sulfuric acid cave open at sea. Fetida Cave represents a unique environment for the study of microbial biogeography and biodiversity in a sulfide-rich aphotic habitat influenced by seawater. In particular, Fetida Cave is constantly subject to the influence of geochemistry and hydrodynamics of the upwelling of sulfidic fluids, inside the cave, and of marine water, entering the cave from the outside opening. In correspondence of the in-cave sulfidic spring inlet, subaerial and submerged environments were featured by more constant physico-chemical parameters and higher concentration of H_2_S and temperatures (slightly thermal) compared to the cave entrance. The host rock (limestone) dissolution is still an active process as testified by the values of the saturation indices revealed in the water pool in particular in the inner portion of Fetida Cave.

### Morphology and distribution of biodeposits from Fetida Cave

Specific inner morphologies and mineralogy associated with sulfuric acid speleogenesis have been previously reported in Santa Cesarea Terme caves by D’Angeli et al. [17]. In particular, abundant microbial biofilms/deposits were visible in the water pool and on the walls and ceilings of the inner zone of Fetida Cave (FC), suggesting that the development of resident microbial communities was closely related to the rising sulfidic fluids and H_2_S degassing.

The water biofilms visible in FC have a morphology similar to that described in other sulfidic cave water streams [12, 22, 52]. However, FC water biofilm pattern was not constant over the year and, generally, biofilms were less thick and dense as compared to the distribution of similar biofilms described in other sulfidic caves [22]. This is due to the fact that Fetida Cave is subject to seawater hydrodynamics which dilutes and occasionally washes the water biofilms away during exceptional tides or high waves.

Unlike the biodeposits covering the cave walls and ceilings, water biofilms were also present at the entrance of FC. They had a prevalent morphology of thin filaments attached to the submerged rocks on the sides of the water stream (named in this work as F-stream), whereas the water filamentous biofilms inside the cave were generally thicker, more abundant and floating on the stream or deposited at the bottom (named F-float or F-sed) (Fig 1). The development of F-stream was probably associated with specific environmental conditions occurring in the most external cave zone in association to i) the physico-chemical gradient created along the cave by the mixture of the warm sulfidic fluid with the marine water and ii) the water turbulence due to the higher exposure to the seawater currents. The microbial communities of F-stream showed important differences respect to F-sed and F-float and these are discussed below.

Abundant vermiculations covered the walls of the inner zone of FC which were featured by irregular morphology, mainly spotted shape, and different colors (i.e. light and dark grey, light and dark brown, red and black), being the grey and brown vermiculations the most abundant. The coloration was not apparently related to specific regions inside the cave, however, the vermiculations with the same aspect were grouped in clusters of different dimensions.

The moonmilk deposits were visible as bright white microcrystalline assemblages of gypsum. They are distinguished from gypsum crusts (that were also present in Fetida Cave and often surrounded the moonmilk deposits) because of their soft texture that resembles toothpaste. Moonmilk deposits were less frequent than vermiculations on the Fetida Cave walls and ceilings; further, whereas vermiculations mainly developed on vertical areas of the cave, moonmilk deposits were mainly visible on the lower side of protruding rocks mostly capturing acidic vapors. As they are intimately attached to the host rock, their development seems associated with carbonate dissolution and gypsum replacement. Indeed, their sampling required carving in the rock, whereas vermiculations were exposed and easy to sample.

### Biodiversity in Fetida Cave water filaments

Despite the high variability of the cave water stream due to the fluctuation in geochemistry and hydrodynamics associated with the seawater and possible alternative organic carbon sources, a high presence of members related to sulfur metabolism and belonging to Gammaproteobacteria, Deltaproteobacteria and Epsilonproteobacteria were revealed in FC water biofilms. These microbial sequences were affiliated with those retrieved from deep-marine environments close to gas seeps (hydrothermal deep vents and cold seeps), and at a lower degree, from microbial mats and biofilms described in other sulfidic caves, these last characterized by more stable conditions and the presence of freshwater streams [52–54].

Gammaproteobacteria of the Thiotrichales and Arenicellales orders dominated the FC filaments. The presence of these microbial groups, especially Thiotrichales, supports the filamentous and web structures of F-stream observed through microscopy, similar to that previously described for water biofilms collected from other cave environments [54]. Arenicellales order has been recently defined [55] and mainly included marine bacteria, some of them isolated from deep-marine environments. While the present study on FC is the first study describing members of Arenicellales order being associated with a marine sulfidic cave microbiology, filamentous sulfur-oxidizing members of Thiotrichales (of Thiotrichaceae family) were found to dominate microbial communities of water biofilms collected from the sulfidic Frasassi Cave [52]. Most of the Arenicellales- and Thiotrichales-related sequences were classified only up to family level (Fig 7). Interestingly, the recently described ‘*Candidatus* Thiopilula’ was dominant in the sedimented biofilms (F-sed-2) (S6 Table, S1 Appendix). ‘*Ca*. Thiopilula’ was previously identified in oxygen minimum zone sediments and cold seeps through metagenomic approaches. The possible contribution of ‘*Ca*. Thiopilula’ in chemolithotrophic processes was supported by transcriptomic results indicating its sulfur oxidation and nitrogenous compounds reduction abilities in microbial mats collected from a deep cold seep [56]. Additional Gammaproteobacteria genera retrieved from the FC water filaments belonged to Oceanospirillales or Chromatiales orders including *Marinobacterium*, *Thiohalophilus*, *Granulosicoccus*, *Halothiobacillus* and *Sedimenticola*. Members of these genera are typically associated with marine and halophilic water habitats, featured by chemolithotrophic activities related to sulfur and sulfidic compounds oxidation, and nitrogen metabolism in anoxia and absence of light [57–60]. In association with chemolithotrophs, FC filaments host marine oligotrophic Gammaproteobacteria Cellvibrionales and Alteromonadales members, the latter being considered dominant colonizers of marine biofilms able to metabolize various hydrocarbon compounds [61], which are also possibly involved in nitrogen and sulfur metabolism in shallow-water hydrothermal vent ecosystems [62].

Epsilonproteobacteria have been described to provide the main form of primary productivity in aphotic sulfur-driven microbial ecosystems, including cave sulfidic springs [63, 64]. Unlike filamentous microbial mats described in other sulfidic caves (e.g. Lower Kane Cave), Epsilonproteobacteria were not the dominant microbial group in Fetida Cave water biofilms, although their abundance increased by moving from the cave entrance towards the inner zone. The higher presence of Epsilonproteobacteria in the inner zone of the cave can be related to their enrichment occurring at low oxygen tension and high H_2_S concentration [10, 22]. We can hypothesize that, even though the general sulfide to oxygen ratio conditions and the seawater-related organic source negatively influence Epsilonproteobacteria growth, the environmental conditions in the inner zone of the cave can sustain their increase in the filament microbial communities. Indeed, in the cave inner zone the H_2_S arises and the slowly flowing water limits oxygen diffusion and hosts deposited mats/filaments. Epsilonproteobacterial sequences in FC were exclusively related to Helicobacteraceae and Campylobacteraceae families, which were mostly represented by *Sulfurimonas*, *Arcobacter*, *Campylobacter* and *Sulfurovum* genera, which are featured by sulfur- and sulfide-oxidizing activities associated with different freshwater and marine environments, including oil fields [65–67].

Besides the increase of sulfur-oxidizing Epsilonproteobacteria sequences, filaments inside the FC showed a higher concentration of the Deltaproteobacteria, Chloroflexi and Deferribacteres as compared to F-stream with a parallel decrease of the marine-associated taxa Actinobacteria, Acidobacteria, Planctomycetes and Alphaproteobacteria [68]. This can be due to the selection imposed on the microbial diversity by the peculiar geo-physical-chemistry of the water inside the cave in relation to the higher concentration of H_2_S, the slower water flow and the absence of light. Among these, *Deltaproteobacteria* are known to include most of the sulfate reducers detected in sulfuric acid caves [69]. At genus level, members of *Desulfocapsa*, *Desulfobulbus*, *Desulfuromusa* and *Desulfovibrio* were found in Fetida Cave (S1 Appendix), which are all known sulfur-reducers that are able to use the organic carbon released by sulfur-oxidizing bacteria and other primary producers, as the electron donors [70]. The physical association between sulfur-oxidizing bacteria (of Gamma- and Epsilonproteobacteria) and *Deltaproteobacteria* has been frequently observed in microbial mats developing in marine and lacustrine sediments but also in other sulfuric acid caves, i.e. Frasassi and Acquasanta Terme, and it has been interpreted as a way to optimize microbial cooperation in the sulfur cycling [52].

Other syntrophic cooperation involving Deltaproteobacteria in FC water filaments, might include members of Anaerolineales order and *Caldithrix* genus, which were previously found to be associated with this proteobacterial class in deep-marine sediments, probably contributing to chemoorganotrophic metabolisms under sulfate reducing conditions and metal reducing and oxidizing processes [71, 72].

### Biodiversity in Fetida Cave vermiculations

Fetida Cave hosts a peculiar type of vermiculation that has been previously named “biovermiculation” [12, 16], because of their possible biological origin and the inclusion of highly diversified and active microbial populations [20]. The biovermiculations typically develop in sulfidic caves and present complex and highly diversified geometric forms resembling carbonic-acid caves vermiculations, although they lack significant clay content [17, 20, 73]. Instead, the mineralogy of vermiculations from FC showed the abundance of quartz, in addition to either Mg and Fe-rich minerals, in brown vermiculations, or K- and Al-rich minerals in grey vermiculations. The content of nitrogen and organic carbon within FC vermiculations were comparable to those reported by Jones et al. [16] for some biovermiculations, in which the biological origin of the included organic matter was demonstrated through isotopic analysis. In line with this, we have found that the microbial communities from the same type of deposits from Fetida Cave included chemolithotrophic microbial taxa previously associated with acidophilic and extreme metal-rich environments and mine tailings, wastewater habitats, activated sludges, marine environments and cave settings (Fig 11, S7 Table, S1 Appendix). In particular, the microbial communities in all the vermiculations under analysis showed microbial populations possibly involved in the nitrogen cycle, i.e. members of Rhodobacterales, Rhodospirillales, Nitrospirales orders and Planctomycetaceae family [58, 74], and in sulfur-reduction and -oxidation under acidophilic conditions and metal-rich environments, i.e. members of Desulfurellaceae, Hydrogenophylaceae, and Acidiferrobacteraceae families [75–78]. Gemmatimonadetes phylum, which is abundant in FC vermiculations, also includes potential sulfate reducing members as revealed by recent genomic analyses [79].

In particular, chemosynthetic processes can be associated with *Nitrospira* of Nitrospirales and to *Sulfurifustis* of Acidiferrobacterales, which are abundant genera in all FC vermiculation samples and are able to perform carbon fixation in association with ammonia- or sulfur-oxidation, respectively. *Nitrospira* members are able to catalyze the complete oxidation of ammonia via nitrite to nitrate [80] and, unlike canonical ammonia-oxidizers, can grow under microaerophilic conditions, providing competitive advantage in nutrient-limited conditions and under biofilm growth, similar to vermiculation conditions [80, 81]. Members of Acidiferrobacterales are able to gain energy from iron oxidation and to use not only O_2_ but also Fe^3+^, NO_3_^−^ as electron acceptor, this being in line with the high concentration of Fe ions and hematite minerals detected in some biovermiculations, mainly in the brownish (V-brown) samples (Table 2). Among these, *Sulfurifustis* strains were predicted to have a certain level of metabolic flexibility, due to the redundancy of genes involved in sulfur oxidation and inorganic carbon fixation [82]. *Sulfurifustis*-related sequences from Fetida Cave are phylogenetically related with sequences retrieved from Frasassi Cave vermiculations, classified as *Acidithiobacillus*, and water streamers, classified as *Sulfurovum*-like, which were associated with possible biomineralization processes [16, 22].

Lastly, in Fetida Cave vermiculations, chemoorganotrophs adapted to oligotrophic or contaminated environments were also detected such as members of Gaiellaceae, Blastocatellaceae and Anaerolinaceae families [83, 84], in addition to copiotrophic bacteria (able to metabolize a wide array of carbon sources) belonging to Sphingobacteriales, Cytophagales and Chlamydiales orders, which were found in diverse terrestrial, aquatic and also underground habitats [85–87].

Members of the main chemolithotrophic and chemoorganotrophic taxa composing Fetida Cave (FC) vermiculations, i.e. Betaproteobacteria, Gammaproteobacteria, Acidobacteria, Planctomyces and Nitrospirae, were previously identified in biovermiculations from Frasassi Cave and Cueva de Villa Luz, indicating that this type of biofilm contains a core set of bacterial phyla which could have synergistic activities.

On the other hand, at lower taxonomy levels, specific bacterial groups distinguished vermiculation samples, even collected from the same cave. In FC, members of sulfur-oxidizing autotroph *Sulfuriferula* genus (of Hydrogenophylales order), abundant in microbial consortia responsible for the weathering of sulfide minerals occurring under acidic conditions [88, 89], and members of the highly adaptable chemoorganotroph *Pseudomonas* genus were present in the two V-grey samples, the first being predominant in V-grey-1 and in traces in V-grey-2, and the second being predominant in V-grey-2 and at 1% in V-grey-1. While Hydrogenophilaceae were present also in the brown vermiculations (although at <1%), Pseudomonadaceae were totally absent. The biovermiculation variability in terms of morphology, organic matter content and microbial composition might reflect various conditions of moisture, condensation exposure and geochemistry of the host rock featuring the different cave wall microniches that need to be further explored.

In a previous work, the biodiversity associated with Frasassi Cave biovermiculations was higher than that identified in the white filaments collected from the water stream in the same cave system [16]. This is not true in FC, where most probably the constant mixture of external seawater with the sulfidic fluid leads to the development of microbial communities (associated with F samples) characterized by the highest biodiversity among the analysed FC biofilms (Fig 4). Nevertheless, FC vermiculations are featured by unusually high biodiversity (having 18 phyla accounting for >1%), in consideration of the oligotrophic habitat provided by the cave wall. The corresponding SEM imaging showed intricate webs and filamentous microbial formation embedded in an irregular extracellular matrix (Fig 3). In this context, processes of *in situ* sediment particles entrapment and organic matter production can create a breeding ground for biovermiculation formation and the development of complex indigenous microbial communities [16].

### Biodiversity in gypsum moonmilk deposits

Moonmilk is a generic term for a soft, wet, pasty texture material with white, grey or yellowish coloration, it generally consists of microcrystalline aggregates of carbonate precipitates with high water content and is present on the walls of many caves under diverse climatic conditions [32, 90, 91]. Fetida Cave (FC) hosts a peculiar type of moonmilk deposit made of gypsum, having bright white coloration, and, unlike the alkaline calcite moonmilk, with an extremely acidic pH, close to 0–1 (Fig 2, Table 2). This type of deposit has been previously observed in SAS systems, without being microbiologically characterized [29].

FC’s gypsum moonmilk presents low diversity microbial communities, which are strongly dominated by only one archaeal genus i.e. *Thermoplasma*, in some cases associated with *Ferroplasma*, the latter present at lower level (Fig 7, S8 Table, S1 Appendix). Both *Ferroplasma* and *Thermoplasma* are cell-wall lacking extremely acidophilic archaea with oligotrophic lifestyles and possible capacities to gain energy by sulfur respiration and iron oxidation. While only few isolates have been characterized, sequences related to these genera have been frequently retrieved from acidophilic and metal-rich environments [25, 92–94]. Few other bacterial taxa, mostly facultative chemolithotrophic, were present in gypsum moonmilk microbial communities i.e. *Acidithiobacillus*, *Metallibacterium*, *Acidibacillus* and *Sulfobacillus*, which are known to be adapted to extremely acidic pH and/or metal-rich environments, some of them being possibly involved in the sulfur cycle and iron-oxidation processes [95]. The high proportion of oligotrophic archaeal populations in gypsum moonmilk with low total C and N values (Table 2) and lower numbers of prokaryotic primary producers (i.e. *Acidithiobacillus* spp.) is an interesting result that guides future metagenomic studies for the identification of key metabolic functions in the Thermoplasmata population.

The microbial community composition of gypsum moonmilk from FC strongly differs from that described in calcite moonmilk deposits, whose biodiversity is mainly characterized by members of aerobic chemoorganotrophic and facultative chemolithotrophs belonging to Alpha-, Beta- and Gamma-proteobacteria and Actinobacteria involved in nitrogen and hydrogen oxidation and with optimal growth at circumneutral pH [96, 97]. On the other hand, the microbial composition of Fetida’s moonmilk showed high similarities with the biodiversity described in acidic pendulous biofilms, named snottites collected from other sulfuric acid caves, i.e. Frasassi and Lower Kane caves [23, 25]. In particular, not only the dominant *Thermoplasma*-related sequences but also most of the abundant SVs within the FC gypsum moonmilk showed highly similarity with those retrieved from Frasassi Cave snottites (S8 Table). This is probably due to the extremely acidic pH and the similar sulfuric acid cave setting and mineralogy shared by the two types of biofilms. On the other hand, gypsum moonmilk and snottites are featured by relevant differences in morphology and arrangement. In fact, gypsum moonmilk is a soft creamy white deposit composed of gypsum microcrystals, which develops on the overhanging exposed walls and ceiling of Fetida Cave from 1 m above the water table, whereas snottites are pendulous structures, developed on gypsum substrate, thriving from 0.5 to 4 m above the water stream. The different morphology and arrangement of these two deposits might be associated with the stability of geochemical and physical-chemical parameters and environmental conditions featuring the hosting caves (e.g. degassing H_2_S and O_2_ content as a function of hydrodynamic conditions, condensation phenomena, possible meteoric water infiltration). These morphological differences might support the difference found in the dominance of specific bacterial taxa, which are affiliated to archaeal oligotrophic *Thermoplasma* genus in the Fetida’s moonmilk, whereas they are affiliated to gammaproteobacterial chemolithoautotrophic *Acidithiobacillus* in Frasassi’s snottite [23, 24]. Probably, the extreme acidophilic community, which is colonizing the moonmilk deposits of Fetida Cave, contributes to the precipitation of gypsum crystals, but further investigations are required to confirm this theory.

## Conclusions

**Fig 14:**
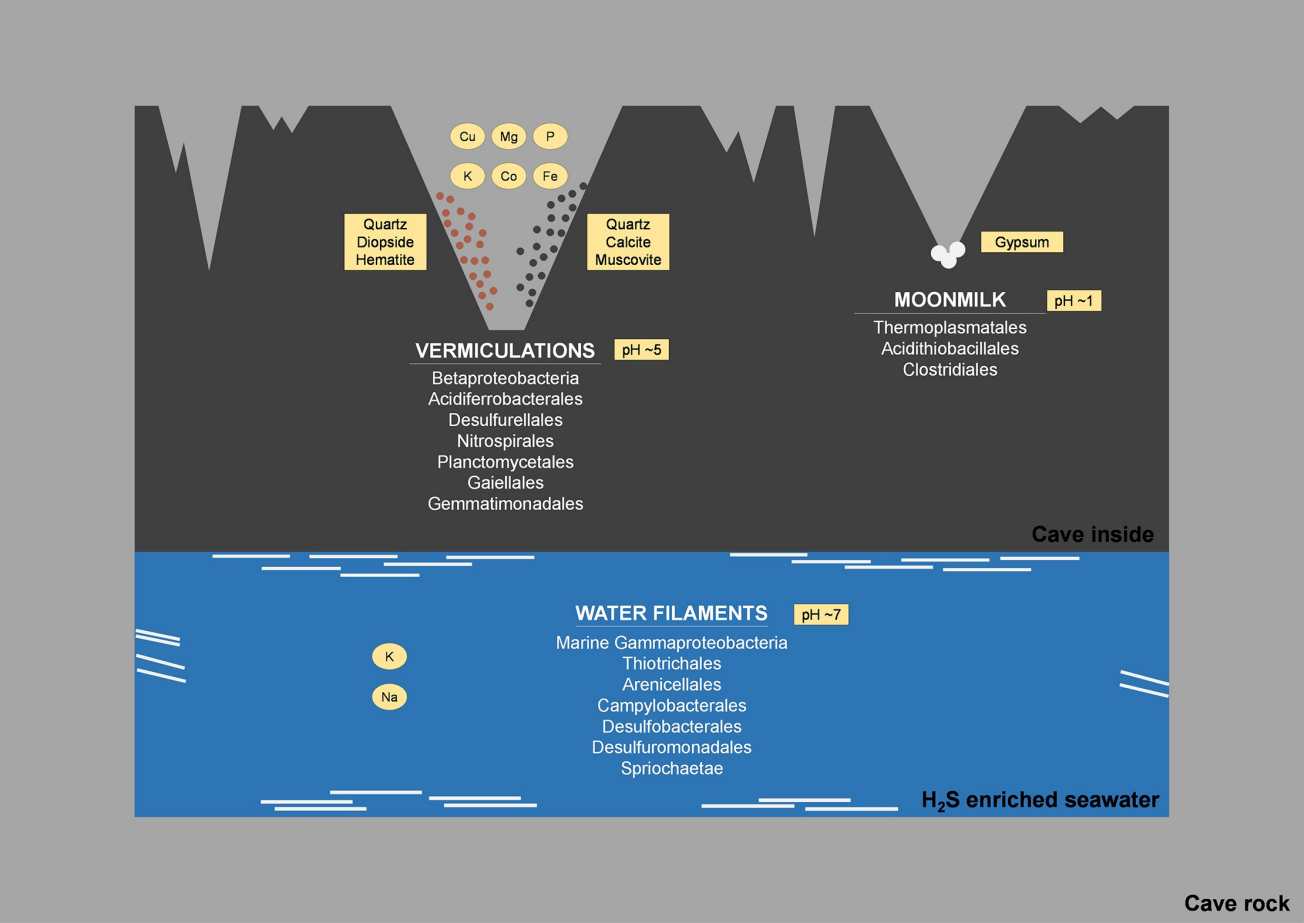
Schematic representation of the distribution pattern of water filaments, (bio)vermiculations and gypsum moonmilk in Fetida Cave, along with the most representative microbial groups (in white) and the main geochemical (within squares) and mineralogical (within ovals) characteristics in each type of biofilm/deposit. The pH values are also reported. For the water filaments, the three filament morphologies are shown, i.e. floating, sedimented, and streamer filaments.

Fetida Cave represents a unique sulfuric acid cave influenced by seawater hydrodynamics, in which rising sulfidic fluids mix with seawaters. This peculiar subterranean environment shows a variety of ecological niches that host different microbial-rich deposits and biofilms floating or deposited in the water (white filaments) or growing on the walls (vermiculations and moonmilk deposits). Each biodeposit is characterized by specific microbial taxa (Fig 14), which are selectively enriched in each of the microbial communities growing as water filaments, vermiculation or in gypsum moonmilk deposits. This is related to the selection imposed by environmental factors linked to the cave environment in which each biodeposit develops, which at various levels include the following aspects: the type of substrate (i.e. the cave wall and ceiling rock or cave water), the pH (extremely acidic in moonmilk, slightly acidic in vermiculations and neutral in water filaments), the amount of condensation on the cave walls and ceiling (i.e. high condensation for the moonmilk development), the amount and type of nutrient input (higher organic carbon is likely in the water stream which is constantly mixed with seawater entering from the coastline), the type of metal and mineral exposure (gypsum for moonmilk, Fe- or Al-containing minerals in vermiculations). In particular, the three biodeposits showed the presence of diverse chemolithotrophic bacterial and archaeal members, which are affiliated with microorganisms retrieved from other sulfuric acid caves and acidic environments, in the case of the biodeposits on the walls. On the other hand, the most representative bacterial taxa of water filaments were affiliated with microorganisms from anoxygenic marine habitats, mainly influenced by gas seepage. Moreover, some correlations could be identified between the microbial composition and specific features of each type of biodeposits, such as the collection site and the morphology of water filaments, the color of vermiculations. Future works will attempt to clarify the role of the microbial populations in each type of deposits in the processes of precipitation of secondary minerals, sulfur cycle, trapping and binding activities, and dissolution/corrosion of rocks.

## Supporting information

S1 Fig Relation between S^2-^ (mg/L) and temperature (°C) of water collected in Fetida Cave. The graph shows a slight tendency of warmer solutions to contain higher S^2-^ concentrations. A1 and A2 were collected at the cave entrance, while B1 and B2 were collected in the cave inner zone.
(TIF)

S2 Fig Geological classification and saturation indices of the Fetida Cave waters as compared to the seawater. A) Ludwig-Langelier diagram showing that all the waters collected at the cave entrance (A samples), in the inner cave zone (B samples) and along the coastline (seawater) clustered in the Na-Cl-SO_4_ sector; B) Mean values of the calcite (C-SI), dolomite (D-SI), and gypsum (G-SI) saturation indices. Dashed line corresponds to the equilibrium state, the points above this state indicate oversaturated waters, whereas the points below indicate undersaturated waters.
(TIF)

S3 Fig Rarefaction curves. Rarefaction analysis of the biofilms collected from Fetida Cave.
(TIF)

S4 Fig Venn diagrams and Sequence Variants (SVs) shared by the different biofilm samples collected from Fetida Cave. The taxonomy classification of the SVs shared by the samples are also indicated in the different tables.
(TIF)

S1 Table Description of the different biofilm samples collected from Fetida Cave. (DOCX)

S2 Table Physico-chemical analyses of Fetida Cave atmosphere. (DOCX)

S3 Table Composition of the waters collected inside the cave and along the coastline (seawater). (DOCX)

S4 Table Trace elements in water samples collected at the Fetida cave entrance, in the deep part of the cave and along the coastline. (DOCX)

S5 Table Summary of Illumina MiSeq sequencing and DADA2 analysis. (DOCX)

S6 Table Most abundant SVs in the white filaments from Fetida Cave. (DOCX)

S7 Table Most abundant SVs in the vermiculations from Fetida Cave. (DOCX)

S8 Table Most abundant SVs in the moonmilk from Fetida Cave. (DOCX)

S1 File Low abundant microbial phyla in Fetida Cave water filaments, vermiculations and moonmilk deposits. The microbial phyla included in this list have abundance <1% in all the analyzed microbial communities and are represented in Fig 6 as “Others”.
(PDF)

S1 Appendix Microbial community composition (at all taxonomy levels) of the samples collected from Fetida Cave as representative of water filaments, vermiculations and moonmilk deposits. (XLSX)
