# Supplementary figures and images for: Geomicrobiology of a seawater-influenced active sulfuric acid cave
Source: PLoS One. 2019 Aug 8;14(8):e0220706. doi: 10.1371/journal.pone.0220706 (PMC6687129; doi:10.1371/journal.pone.0220706)

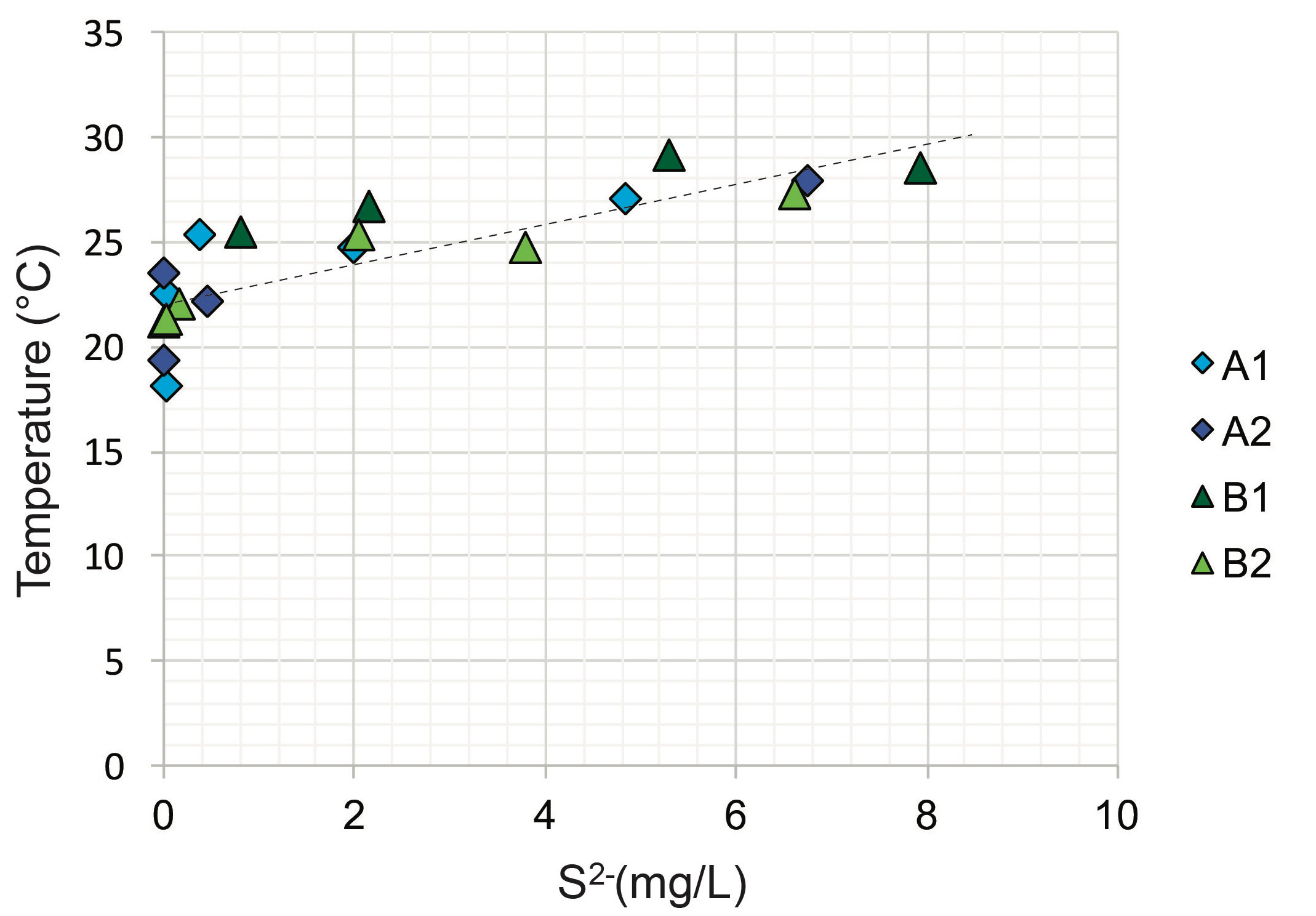

Supplement: S1 Fig — The graph shows a slight tendency of warmer solutions to contain higher S2- concentrations. A1 and A2 were collected at the cave entrance, while B1 and B2 were collected in the cave inner zone. (TIF) [file pone.0220706.s001.tif]

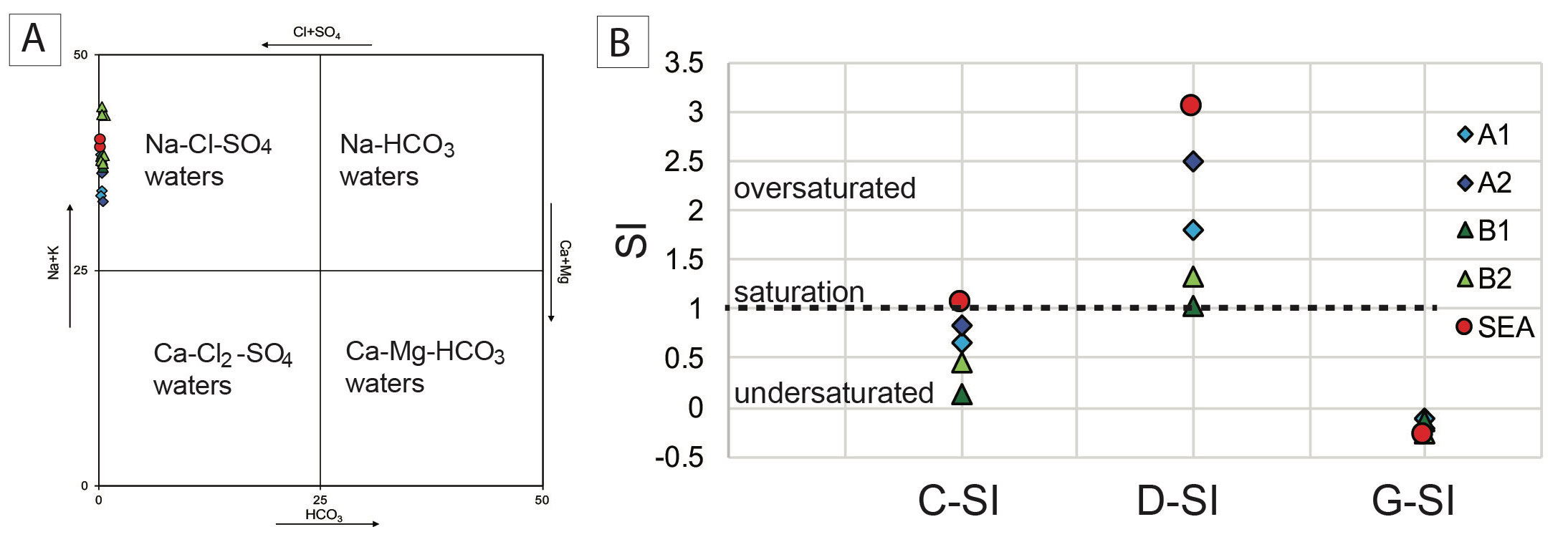

Supplement: S2 Fig — A) Ludwig-Langelier diagram showing that all the waters collected at the cave entrance (A samples), in the inner cave zone (B samples) and along the coastline (seawater) clustered in the Na-Cl-SO4 sector; B) Mean values of the calcite (C-SI), dolomite (D-SI), and gypsum (G-SI) saturation indices. Dashed line corresponds to the equilibrium state, the points above this state indicate oversaturated waters, whereas the points below indicate undersaturated waters. (TIF) [file pone.0220706.s002.tif]

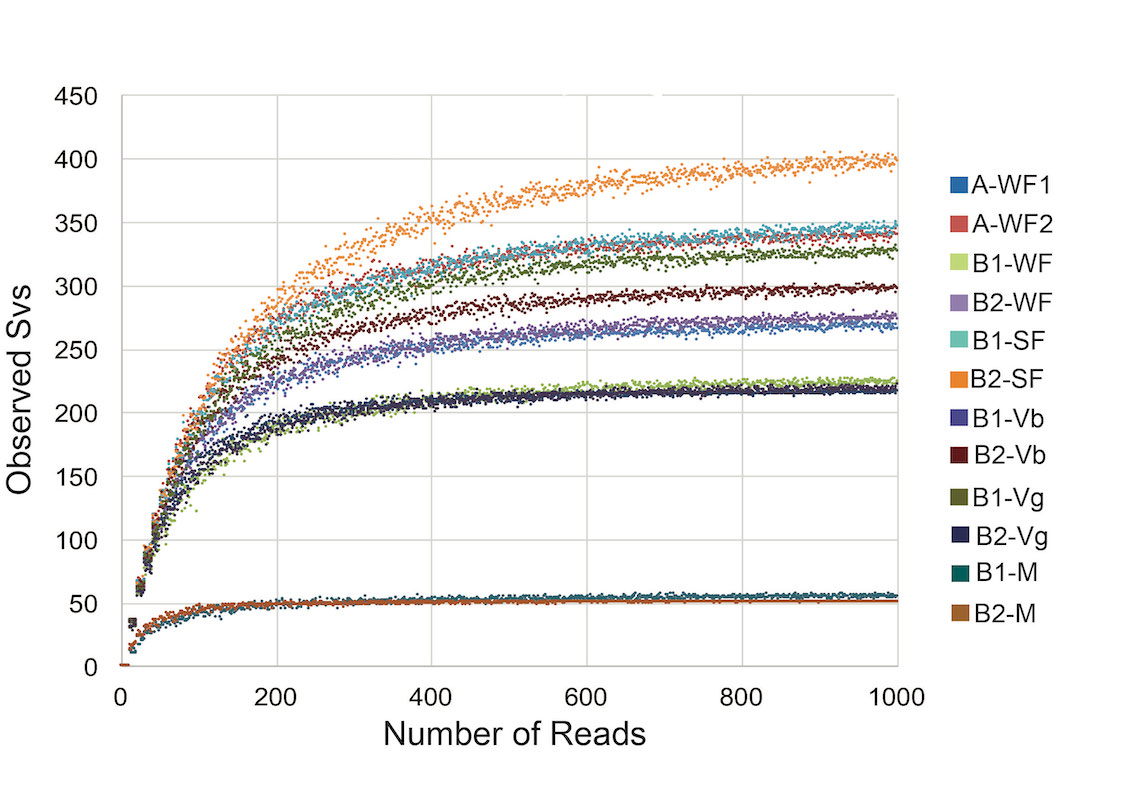

Supplement: S3 Fig — Rarefaction analysis of the biofilms collected from Fetida Cave. (TIF) [file pone.0220706.s003.tif]

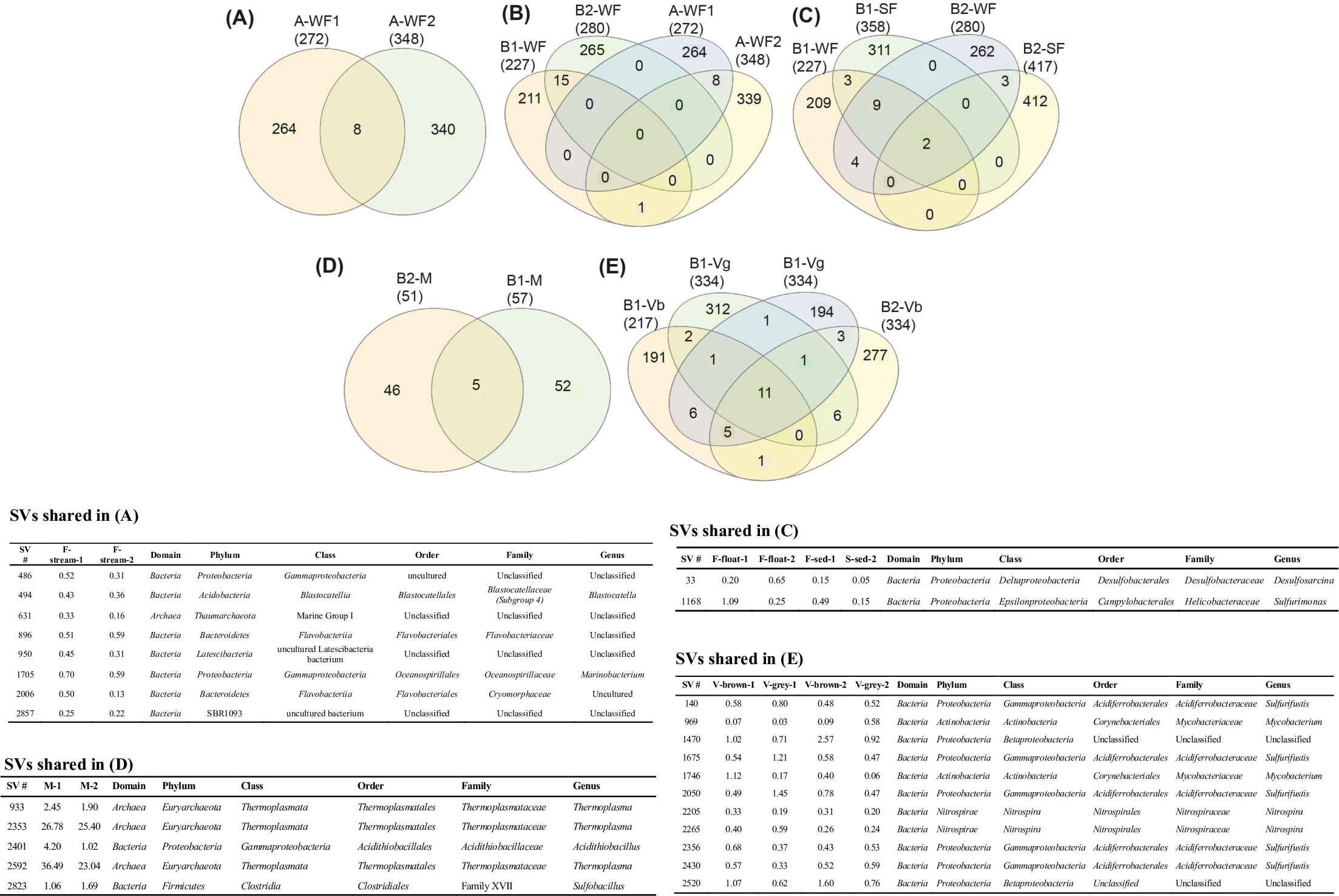

Supplement: S4 Fig — The taxonomy classification of the SVs shared by the samples are also indicated in the different tables. (TIF) [file pone.0220706.s004.tif]
