# Supplementary material for: Geomicrobiology of a seawater-influenced active sulfuric acid cave
Source: PLoS One. 2019 Aug 8;14(8):e0220706. doi: 10.1371/journal.pone.0220706 (PMC6687129; doi:10.1371/journal.pone.0220706)
Supplement: S1 Table — (DOCX) [file pone.0220706.s005.docx]

**S1 Table. Description of the different biofilm samples collected from Fetida Cave**

| **Sample name** | **Cave Site** | **Description** |
| --- | --- | --- |
| F-stream-1 | Entrance | White water filaments attached at the side walls in a squared water pool close to the cave entrance |
| F-stream-2 | Entrance | White water filaments attached at the side walls of the water stream close to the cave entrance |
| F-float-1 | Inner zone | White water filaments floating on the water table close to the rising H_2_S-rich fluids location |
| F-float-2 | Inner zone | White water filaments floating on the water table in the cave inner zone |
| F-sed-1 | Inner zone | Sedimented white filaments at the bottom of the water stream close to the rising H_2_S-rich fluids location |
| F-sed-2 | Inner zone | Sedimented white filaments at the bottom of the water stream in the cave inner zone |
| V-brown-1 | Inner zone | Brown vermiculation taken from the wall at 1.60 m height from the boardwalk close to the rising H_2_S-rich fluids location |
| V-brown-2 | Inner zone | Brown vermiculation taken from the wall at 1.50 m height from the boardwalk in the cave inner zone |
| V-grey-1 | Inner zone | Grey vermiculation deposit collected on the wall at 1.90 m height from the boardwalk close to the rising H_2_S-rich fluids location |
| V-grey-2 | Inner zone | Grey vermiculation taken from the wall at 1.70 m height from the boardwalk in the cave inner zone |
| M-1 | Inner zone | Moonmilk deposit sampled on the wall at 1.70 m height from the boardwalk close to the rising H_2_S-rich fluids location |
| M-2 | Inner zone | Moonmilk deposit sampled from the wall at 1.70 m height from the boardwalk in the cave inner zone |
