# Supplementary material for: Geomicrobiology of a seawater-influenced active sulfuric acid cave
Source: PLoS One. 2019 Aug 8;14(8):e0220706. doi: 10.1371/journal.pone.0220706 (PMC6687129; doi:10.1371/journal.pone.0220706)
Supplement: S2 Table — (DOCX) [file pone.0220706.s006.docx]

**S2 Table. Physico-chemical analyses of Fetida Cave atmosphere**

|  |  | **T (°C)** | **O_2_ (%)** | **SO_2_ (µM)** | **H_2_S (mg/L)** |
| --- | --- | --- | --- | --- | --- |
| Air _entrance_ | Min | 15.63 | 20.80 | 0.00 | 0.00 |
|  | Max | 26.08 | 20.80 | 1.57 | 4.1 |
|  | **Mean** | **21.42** | **20.80** | **0.73** | **0.3** |
|  | Standard deviation | 2.33 | 0.00 | 0.78 | 0.34 |
| Air _inner zone_ | Min | 20.51 | 20.80 | 0.00 | 0.6 |
|  | Max | 24.72 | 20.80 | 1.56 | 15.4 |
|  | **Mean** | **22.76** | **20.80** | **0.63** | **2.61** |
|  | Standard deviation | 0.53 | 0.00 | 0.76 | 2.14 |
