# Supplementary material for: Geomicrobiology of a seawater-influenced active sulfuric acid cave
Source: PLoS One. 2019 Aug 8;14(8):e0220706. doi: 10.1371/journal.pone.0220706 (PMC6687129; doi:10.1371/journal.pone.0220706)
Supplement: S3 Table — (DOCX) [file pone.0220706.s007.docx]

**S3 Table Composition of the waters collected inside the cave and along the coastline (seawater).^a^**

|  | | **T (°C)** | **pH** | **S^2-^ (mg/L)** | **Na (mg/L)** | **K (mg/L)** | **Ca (mg/L)** | **Mg (mg/L)** | **Cl^-^ (mg/L)** | **SO_4_^2-^ (mg/L)** | **HCO_3_^-^ (mg/L)** | **F^-^ (mg/L)** | **Br^-^ (mg/L)** | **NO_3_^-^ (mg/L)** | **TDS (g/L)** |
| --- | --- | --- | --- | --- | --- | --- | --- | --- | --- | --- | --- | --- | --- | --- | --- |
| Cave entrance 1^b^ | m^c^ | 18.1 | 7.02 | 0.02 | 6012.0 | 329.2 | 507.8 | 824.6 | 12873.9 | 2135.3 | 163.89 | 1.3 | 58.6 | 0.0 | 17.21 |
|  | M | 27.1 | 8.16 | 4.84 | 9436.0 | 654.4 | 993.3 | 1321.4 | 20348.1 | 2914.5 | 338.52 | 4.0 | 81.0 | 44.7 | 26.06 |
|  | **a** | **23.2** | **7.41** | **1.44** | **7616.3** | **422.7** | **700.3** | **1109.5** | **16094.5** | **2610.4** | **206.80** | **2.5** | **68.7** | **32.7** | **22.58** |
|  | me | 23.9 | 7.34 | 0.37 | 6857.5 | 383.6 | 682.5 | 1177.8 | 16460.1 | 2820.3 | 174.93 | 2.2 | 67.8 | 43.1 | 22.81 |
|  | sd | 3.0 | 0.38 | 2.06 | 1547.2 | 132.7 | 189.6 | 195.8 | 3142.7 | 352.6 | 74.29 | 1.0 | 8.4 | 21.8 | 3.49 |
| Cave entrance 2 | m | 19.3 | 6.97 | 0.01 | 5349.4 | 363.8 | 533.8 | 1106.1 | 9803.7 | 2533.0 | 182.24 | 1.3 | 60.1 | 0.0 | 16.57 |
|  | M | 27.9 | 7.86 | 6.75 | 8117.4 | 450.5 | 618.4 | 1215.6 | 13986.0 | 4195.9 | 185.67 | 4.7 | 139.6 | 36.0 | 20.12 |
|  | **a** | **22.8** | **7.40** | **2.41** | **7010.3** | **405.5** | **571.1** | **1160.7** | **12414.8** | **3537.0** | **183.65** | **3.3** | **87.4** | **16.1** | **18.38** |
|  | me | 22.1 | 7.38 | 0.47 | 7564.0 | 402.2 | 561.1 | 1160.4 | 13454.7 | 3882.3 | 183.05 | 3.9 | 62.4 | 12.3 | 18.45 |
|  | sd | 3.6 | 0.36 | 3.77 | 1464.7 | 43.4 | 43.2 | 54.8 | 2276.8 | 883.6 | 1.79 | 1.8 | 45.3 | 18.3 | 1.774 |
| Cave inner zone 1 | m | 21.20 | 6.76 | 0.01 | 5911.3 | 276.0 | 459.7 | 693.5 | 11126.0 | 1866.6 | 173.29 | 0.7 | 57.5 | 0.0 | 15.00 |
|  | M | 29.1 | 7.20 | 7.92 | 9683.8 | 475.8 | 1013.0 | 1349.0 | 17691.7 | 3673.2 | 220.70 | 3.4 | 132.7 | 38.9 | 23.27 |
|  | **a** | **26.05** | **6.96** | **3.24** | **8204.2** | **383.8** | **708.5** | **987.0** | **14935.2** | **2746.4** | **196.46** | **1.9** | **78.3** | **22.6** | **20.06** |
|  | me | 26.10 | 6.93 | 2.14 | 8568.0 | 381.6 | 723.1 | 968.5 | 15475.1 | 2586.6 | 196.11 | 1.9 | 67.5 | 36.7 | 21.02 |
|  | sd | 2.83 | 0.17 | 3.30 | 1428.1 | 81.2 | 212.6 | 239.7 | 2421.6 | 665.7 | 16.77 | 1.0 | 30.8 | 20.7 | 3.16 |
| Cave inner zone 2 | m | 21.30 | 6.86 | 0.03 | 5110.2 | 175.7 | 280.8 | 287.0 | 8386.6 | 946.6 | 181.95 | 0.6 | 34.8 | 0.0 | 10.41 |
|  | M | 27.3 | 7.30 | 6.61 | 12464.0 | 433.3 | 859.2 | 1317.6 | 20016.9 | 3261.4 | 258.65 | 2.3 | 68.5 | 37.2 | 24.03 |
|  | **a** | **24.19** | **7.10** | **2.53** | **8316.1** | **294.9** | **537.4** | **693.4** | **14107.1** | **2359.6** | **223.14** | **1.5** | **60.7** | **26.3** | **18.30** |
|  | me | 24.80 | 7.12 | 2.06 | 8113.3 | 291.9 | 500.6 | 622.5 | 14375.9 | 2426.7 | 219.23 | 1.6 | 65.5 | 33.0 | 19.22 |
|  | sd | 2.10 | 0.18 | 2.75 | 2739.7 | 92.8 | 199.8 | 345.4 | 4315.8 | 813.1 | 25.71 | 0.8 | 12.8 | 14.1 | 5.06 |
| Sea water | m | 15.7 | 8.18 | BDL | 10573.8 | 451.5 | 459.3 | 1300.6 | 19042.4 | 2905.8 | 162.73 | 2.0 | 0.0 | 44.1 | 24.39 |
|  | M | 21.1 | 8.20 | BDL | 12078.6 | 664.4 | 462.7 | 1337.1 | 21499.9 | 3100.5 | 202.02 | 2.1 | 75.2 | 44.1 | 27.34 |
|  | **a** | **18.9** | **8.19** | **BDL** | **11326.2** | **558.0** | **461.0** | **1318.8** | **20271.1** | **3003.1** | **181.05** | **2.1** | **37.6** | **44.1** | **25.86** |
|  | me | 19.8 | 8.19 | BDL | 11326.2 | 558.0 | 461.0 | 1318.8 | 20271.1 | 3003.1 | 185.06 | 2.1 | 37.6 | 44.1 | 25.86 |
|  | sd | 2.8 | 0.01 | BDL | 1064.1 | 150.5 | 2.4 | 25.8 | 1737.7 | 137.7 | 17.77 | 0.1 | 53.2 | 0.0 | 2.08 |

^a^ Major, minor elements are reported in mg/L (ppm), TDS stands for Total Dissolved Solids and is expressed in g/L.

^b^ Two samples from each cave site (entrance or inner zone) were collected and analyzed.

^c^ m=min, M=max, a=mean, me=median, sd=standard deviation, BDL=below the detection limit
