# Supplementary material for: Geomicrobiology of a seawater-influenced active sulfuric acid cave
Source: PLoS One. 2019 Aug 8;14(8):e0220706. doi: 10.1371/journal.pone.0220706 (PMC6687129; doi:10.1371/journal.pone.0220706)
Supplement: S4 Table — (DOCX) [file pone.0220706.s008.docx]

**S4 Table. Trace elements (in ppb) in water samples collected at the cave entrance, in the deep part of the cave (two samples from each site) and along the coastline (sea water).**

| **Cave sampling site** | **Al** | **V** | **Cr** | **Mn** | **Fe** | **Co** | **Ni** | **Cu** | **Zn** | **Ga** | **As** | **Se** | **Rb** | **Sr** | **Ag** | **Cd** | **Cs** | **Ba** | **Tl** | **Pb** | **Bi** | **U** |
| --- | --- | --- | --- | --- | --- | --- | --- | --- | --- | --- | --- | --- | --- | --- | --- | --- | --- | --- | --- | --- | --- | --- |
| Entrance site 1 | 2.21 | 0.69 | 0.00 | 1.16 | 4.93 | 0.00 | 0.00 | 0.38 | 1.13 | 0.47 | 0.35 | 0.38 | 34.87 | 2155.7 | 0.01 | 0.00 | 0.36 | 8.79 | 0.00 | 0.00 | 0.30 | 0.16 |
| Entrance site 2 | 0.00 | 0.36 | 0.00 | 0.09 | 0.00 | 0.00 | 0.00 | 0.39 | 0.00 | 0.01 | 0.13 | 0.48 | 16.12 | 963.2 | 0.00 | 0.00 | 0.05 | 1.62 | 0.00 | 0.00 | 0.00 | 0.00 |
| Inner zone site 1 | 39.44 | 1.08 | 0.00 | 1.21 | 20.15 | 0.04 | 0.00 | 0.52 | 5.48 | 0.76 | 0.48 | 0.29 | 41.07 | 3017.7 | 0.07 | 0.00 | 0.49 | 10.74 | 0.01 | 0.00 | 0.01 | 0.17 |
| Inner zone site 2 | 21.84 | 1.10 | 0.00 | 0.93 | 14.33 | 0.00 | 0.01 | 1.06 | 2.23 | 0.75 | 0.48 | 0.57 | 32.60 | 2438.8 | 0.00 | 0.00 | 0.22 | 10.40 | 0.00 | 0.01 | 0.02 | 0.09 |
| Sea water | 0.00 | 0.41 | 0.00 | 0.03 | 0.00 | 0.00 | 0.00 | 0.48 | 0.00 | 0.01 | 0.16 | 0.60 | 15.01 | 828.8 | 0.00 | 0.00 | 0.00 | 0.03 | 1.89 | 0.00 | 0.00 | 0.00 |
