# Supplementary material for: Geomicrobiology of a seawater-influenced active sulfuric acid cave
Source: PLoS One. 2019 Aug 8;14(8):e0220706. doi: 10.1371/journal.pone.0220706 (PMC6687129; doi:10.1371/journal.pone.0220706)
Supplement: S5 Table — (DOCX) [file pone.0220706.s009.docx]

**S5 Table. Summary of Illumina MiSeq sequencing and DADA2 analysis**

| **Sample** | **Raw Reads#** | **Filtered Reads#** | **Observed SVs** |
| --- | --- | --- | --- |
| F-stream-1 | 57827 | 11451 | 272 |
| F-stream-2 | 74389 | 15970 | 338 |
| F-float-1 | 62722 | 9689 | 219 |
| F-float-2 | 69993 | 11426 | 274 |
| F-sed-1 | 75923 | 15649 | 347 |
| F-sed-2 | 114750 | 24789 | 398 |
| V-brown-1 | 68415 | 9773 | 216 |
| V-brown-2 | 76295 | 14090 | 298 |
| V-grey-1 | 129163 | 19926 | 324 |
| V-grey-2 | 73840 | 11467 | 218 |
| M-1  M-2 | 95909  55143 | 6577  3729 | 57  51 |
