# Supplementary material for: Geomicrobiology of a seawater-influenced active sulfuric acid cave
Source: PLoS One. 2019 Aug 8;14(8):e0220706. doi: 10.1371/journal.pone.0220706 (PMC6687129; doi:10.1371/journal.pone.0220706)
Supplement: S6 Table — (DOCX) [file pone.0220706.s010.docx]

**S7 Table. Most abundant SVs in the white filaments from Fetida Cave.**

| **SV #** | **F-stream-1** | **F-stream-2** | **F-float-1** | **F-float-2** | **F-sed-1** | **F-sed-2** | **Taxonomy** | **Best Blast Hit** | **Source** | **Accession no.** | **ID%** |
| --- | --- | --- | --- | --- | --- | --- | --- | --- | --- | --- | --- |
| 196 | 0.00 | 0.96 | 0.00 | 0.00 | 0.00 | 0.00 | *p_Acidobacteria; o_Subgroup 10* | Uncultured prokaryote New.ReferenceOTU347 | Marine intertidal outcrops | KT978987 | 98.29 |
| 402 | 0.00 | 0.00 | 0.00 | 0.00 | 0.00 | 1.43 | *c_Gammaproteobacteria; g_*Candidatus *Thiopilula* | Candidatus Thiopilula aggregata | Marine sediment | FN811664 | 98.97 |
| 450 | 0.00 | 0.00 | 0.00 | 1.37 | 0.00 | 0.00 | *c_Deltaproteobacteria; g_Desulfuromusa* | Bacterium enrichment culture CavesPI2_11A_27f | Zakynthos submarine caves | MF627415 | 98.63 |
| 885 | 0.00 | 0.00 | 0.00 | 0.00 | 0.79 | 0.00 | *c_Gammaproteobacteria; g_Halomonas* | Uncultured bacterium OTU3 | Oil-polluted saline soil | KY385430 | 99.32 |
| 949 | 0.00 | 0.00 | 0.00 | 1.29 | 0.00 | 0.00 | *c_Epsilonproteobacteria; g_Sulfurimonas* | Uncultured bacterium HL-WM bac37 | Hot Lake hydrothermal vent | HG738998 | 97.26 |
| 1303 | 1.20 | 0.00 | 0.00 | 0.00 | 0.00 | 0.00 | *c_Deltaproteobacteria; f_Desulfobacteraceae* | Uncultured bacterium clone 988325545618 | Thalassohaline lake | MG006044 | 99.66 |
| 1330 | 0.00 | 0.00 | 2.20 | 0.32 | 0.77 | 0.00 | *c_Gammaproteobacteria; f_Arenicellaceae* | Uncultured bacterium MD01a2 9075 | CO_2_ exposed soil | JQ373314 | 98.97 |
| 1543 | 1.23 | 0.00 | 0.00 | 0.00 | 0.00 | 0.00 | *c_Alphaproteobacteria; g_Pelagibus* | Uncultured prokaryote New.ReferenceOTU347 | Marine intertidal outcrops | KT974980 | 97.95 |
| 1564 | 0.00 | 1.04 | 0.00 | 0.00 | 0.00 | 0.00 | *p_Acidobacteria; o_Subgroup 10* | Uncultured prokaryote New.ReferenceOTU347 | Marine intertidal outcrops | KT978987 | 97.95 |
| 1893 | 0.00 | 0.00 | 0.00 | 1.25 | 0.00 | 0.00 | *c_Epsilonproteobacteria; g_Sulfurimonas* | Uncultured bacterium HL-WM bac37 | Hot Lake hydrothermal vent | HG738998 | 97.60 |
| 1933 | 0.00 | 0.00 | 2.12 | 0.38 | 2.64 | 0.00 | *c_Gammaproteobacteria; f_Arenicellaceae* | Uncultured bacterium MD01a2 9075 | CO_2_ exposed soil | JQ373314 | 98.97 |
| 2636 | 0.00 | 0.00 | 4.03 | 0.30 | 0.88 | 0.00 | *c_Gammaproteobacteria; f_Arenicellaceae* | Uncultured bacterium MD01a2 9075 | CO_2_ exposed soil | JQ373314 | 99.32 |
| 2710 | 0.00 | 0.00 | 0.00 | 0.00 | 0.00 | 1.45 | *c_Gammaproteobacteria; g_*Candidatus *Thiopilula* | Candidatus Thiopilula aggregata | Marine sediment | FN811664 | 98.29 |
| 2775 | 0.00 | 0.00 | 0.00 | 0.00 | 0.00 | 4.29 | *c_Gammaproteobacteria; g_*Candidatus *Thiopilula* | Candidatus Thiopilula aggregata | Marine sediment | FN811664 | 98.63 |
| 2880 | 1.04 | 0.00 | 0.00 | 0.00 | 0.00 | 0.00 | *c_Gammaproteobacteria; g_Cocleimonas* | Uncultured bacterium KZNMV-0-B2 | Submarine mud volcano | FJ712400 | 97.60 |

^a^ The table shows the a SVs > 1% at least in one of the white filament samples.

^b^ The SILVA taxonomy is assigned based on a search threshold of 0.8.

^c^ The grey shade differentiates the abundance i.e. black= abundance > 10%. dark grey= abundance>1%. light grey = abundance<1%. white=not detected.
