# Supplementary material for: Geomicrobiology of a seawater-influenced active sulfuric acid cave
Source: PLoS One. 2019 Aug 8;14(8):e0220706. doi: 10.1371/journal.pone.0220706 (PMC6687129; doi:10.1371/journal.pone.0220706)
Supplement: S7 Table — (DOCX) [file pone.0220706.s011.docx]

**S9 Table. Most abundant SVs in the vermiculations from Fetida Cave.^a,b,c^**

| **SV #** | **V-brown-1** | **V-brown-2** | **V-grey-1** | **V-grey-2** | **Taxonomy** | **BBH** | **Source** | **Accession no.** | **ID%** |
| --- | --- | --- | --- | --- | --- | --- | --- | --- | --- |
| 358 | 0.00 | 0.00 | 0.00 | 2.09 | *c_Gammaproteobacteria; g_Pseudomonas* | Uncultured bacterium SPN2000-90day-98 | PAHs contaminated soil | MF314724 | 98.97 |
| 443 | 0.00 | 0.92 | 0.00 | 0.00 | *p_Actinobacteria; g_Pseudonocardia* | Uncultured Pseudonocardia sp. OTU49 SY16 227987 | Coral samples | KY393407 | 97.95 |
| 591 | 0.00 | 0.00 | 3.38 | 0.00 | *c_Betaproteobacteria; g_Sulfuriferula* | Uncultured prokaryote PC08-66-138 | Frasassi stream biofilm | KM410852 | 98.97 |
| 1470 | 1.02 | 2.57 | 0.71 | 0.92 | *c_Betaproteobacteria;* unclassified | Uncultured Comamonadaceae 3d 19492 | Sewage sludge | MG802053 | 96.58 |
| 1675 | 0.54 | 0.58 | 1.21 | 0.47 | *c_Gammaproteobacteria; g_Sulfurifustis* | Uncultured bacterium 36MIC041 | Concrete sewer biofilm | JF341459 | 98.63 |
| 1746 | 1.12 | 0.40 | 0.17 | 0.06 | *p_Actinobacteria; g_Mycobacterium* | Uncultured bacterium NC1F4b510764 | CO_2_ exposed soil | JQ378183 | 99.32 |
| 1773 | 0.00 | 0.00 | 0.00 | 2.93 | *c_Gammaproteobacteria; g_Pseudomonas* | Uncultured bacterium SPN2000-90day-98 | PAHs contaminated soil | MF314724 | 98.63 |
| 1935 | 0.90 | 0.00 | 0.00 | 0.00 | *p_Nitrospirae; g_Nitrospira* | Uncultured bacterium OTU4582 | Forest soil | MH528230 | 98.97 |
| 2050 | 0.49 | 0.78 | 1.45 | 0.47 | *c_Gammaproteobacteria; g_Sulfurifustis* | Uncultured bacterium 36MIC041 | Concrete sewer biofilm | JF341459 | 98.97 |
| 2274 | 0.00 | 0.00 | 0.00 | 2.11 | *c_Gammaproteobacteria; g_Pseudomonas* | Uncultured bacterium SPN2000-90day-98 | PAHs contaminated soil | MF314724 | 98.63 |
| 2520 | 1.07 | 1.60 | 0.62 | 0.76 | *c_Betaproteobacteria;* unclassified | Uncultured Comamonadaceae 3d 19492 | Sewage sludge | MG802053 | 96.23 |
| 2558 | 0.00 | 0.00 | 3.39 | 0.00 | *c_Betaproteobacteria; g_Sulfuriferula* | Uncultured prokaryote PC08-66-138 | Frasassi stream biofilm | KM410852 | 98.97 |
| 2802 | 0.00 | 0.00 | 5.31 | 0.30 | *c_Betaproteobacteria; g_Sulfuriferula* | Uncultured prokaryote PC08-66-138 | Frasassi stream biofilm | KM410852 | 98.97 |

^a^ The table shows the SVs > 1% at least in one of the vermiculation samples.

^b^ The SILVA taxonomy is assigned based on a search threshold of 0.8.

^c^ The grey shade differentiates the abundance i.e. black= abundance > 10%. dark grey= abundance>1%. light grey = abundance<1%. white=not detected.
