# Supplementary material for: Geomicrobiology of a seawater-influenced active sulfuric acid cave
Source: PLoS One. 2019 Aug 8;14(8):e0220706. doi: 10.1371/journal.pone.0220706 (PMC6687129; doi:10.1371/journal.pone.0220706)
Supplement: S8 Table — (DOCX) [file pone.0220706.s012.docx]

**S11 Table. Most abundant SVs in the moonmilk from Fetida Cave.^a,b,c^**

| **SV #** | **M-1** | **M-2** | **Taxonomy** | **Best Blast Hit** | **Source** | **Accession no.** | **ID%** |
| --- | --- | --- | --- | --- | --- | --- | --- |
| 277 | 0.00 | 3.11 | *p_Chlamydiae; f_Simkaniaceae* | Uncultured *Chlamydiae* bacterium 2H69 | Raw source freshwater | GU074172 | 99.64 |
| 312 | 0.00 | 2.52 | *p_Euryarchaeota; g_Thermoplasma* | Uncultured archaeon YTK10 | Acid mine drainage water | MH057131 | 97.60 |
| 497 | 1.82 | 0.00 | *p_Actinobacteria; o_Acidimicrobiales* | Actinobacteria bacterium USS-CCA1 | Abandoned coal mine | MF503098 | 98.63 |
| 933 | 2.45 | 1.90 | *p_Euryarchaeota; g_Thermoplasma* | Uncultured archaeon YTK10 | Acid mine drainage water | MH057131 | 98.29 |
| 2353 | 26.78 | 25.40 | *p_Euryarchaeota; g_Thermoplasma* | Uncultured archaeon RS09a33 | Frasassi acidic snottite | KC582526 | 98.97 |
| 2401 | 4.20 | 1.02 | *c_Gammaproteobacteria; g_Acidithiobacillus* | *Acidithiobacillus thiooxidans* PC30c | Frasassi acidic snottite | KU249609 | 98.63 |
| 2592 | 36.49 | 23.04 | *p_Euryarchaeota; g_Thermoplasma* | Uncultured archaeon RS09a33 | Frasassi acidic snottite | KC582526 | 98.63 |
| 2833 | 0.00 | 3.03 | *c_Gammaproteobacteria; g_Metallibacterium* | Uncultured prokaryote PC08-64-15 | Frasassi stream biofilm | KM410777 | 96.92 |

^a^ The table shows the SVs > 1% at least in one of the moonmilk samples.

^b^ The SILVA taxonomy is assigned based on a search threshold of 0.8.

^c^ The grey shade differentiates the abundance i.e. black= abundance > 10%. dark grey= abundance>1%. light grey = abundance<1%. white=not detected.
