# Supplementary material for: Geomicrobiology of a seawater-influenced active sulfuric acid cave
Source: PLoS One. 2019 Aug 8;14(8):e0220706. doi: 10.1371/journal.pone.0220706 (PMC6687129; doi:10.1371/journal.pone.0220706)
Supplement: S1 File — The microbial phyla included in this list have abundance <1% in all the analyzed microbial communities and are represented in Fig 6 as “Others”. (PDF) [file pone.0220706.s013.pdf]

In Fig 6 “Others” refers to the following bacterial and archeal phyla: AC1, Acetothermia, Aenigmarchaeota, Altiarchaeales, Aminicenantes, Ancient Archaeal Group(AAG), Armatimonadetes, BRC1, Caldiserica, Deinococcus-Thermus, Diapherotrites, Elusimicrobia, Fibrobacteres, Fusobacteria, GAL15, Gracilibacteria, Hydrogenedentes, KSB3 (Modulibacteria), Lentisphaerae, Lokiarchaeota, Marine Hydrothermal Vent Group(MHVG), Marinimicrobia (SAR406 clade), Microgenomates, Nitrospinae, Nitrospinae, Peregrinibacteria, Saccharibacteria, SBR1093, SR1 (Absconditabacteria), Tectomicrobia, Thermotogae, WA-aaa01f12, WS1.
